# Acetyl-CoA is a key molecule for nephron progenitor cell pool maintenance

**DOI:** 10.1038/s41467-023-43513-7

**Published:** 2023-11-25

**Authors:** Fabiola Diniz, Nguyen Yen Nhi Ngo, Mariel Colon-Leyva, Francesca Edgington-Giordano, Sylvia Hilliard, Kevin Zwezdaryk, Jiao Liu, Samir S. El-Dahr, Giovane G. Tortelote

**Affiliations:** 1 Section of Pediatric Nephrology, Department of Pediatrics, Tulane University School of Medicine, New Orleans, LA 70112 USA; 2 Department of Microbiology and Immunology, Tulane University School of Medicine, New Orleans, LA 70112 USA; 3 Department of Human Genetics, Tulane University School of Medicine, New Orleans, LA 70112 USA

**Keywords:** Cell growth, Nephrons, Biochemistry, Molecular medicine

## Abstract

Nephron endowment at birth impacts long-term renal and cardiovascular health, and it is contingent on the nephron progenitor cell (NPC) pool. Glycolysis modulation is essential for determining NPC fate, but the underlying mechanism is unclear. Combining RNA sequencing and quantitative proteomics we identify 267 genes commonly targeted by Wnt activation or glycolysis inhibition in NPCs. Several of the impacted pathways converge at Acetyl-CoA, a co-product of glucose metabolism. Notably, glycolysis inhibition downregulates key genes of the Mevalonate/cholesterol pathway and stimulates NPC differentiation. Sodium acetate supplementation rescues glycolysis inhibition effects and favors NPC maintenance without hindering nephrogenesis. Six2Cre-mediated removal of ATP-citrate lyase (*Acly*), an enzyme that converts citrate to acetyl-CoA, leads to NPC pool depletion, glomeruli count reduction, and increases *Wnt4* expression at birth. Sodium acetate supplementation counters the effects of *Acly* deletion on cap-mesenchyme. Our findings show a pivotal role of acetyl-CoA metabolism in kidney development and uncover new avenues for manipulating nephrogenesis and preventing adult kidney disease.

## Abstract (web summary)

Cell metabolism plays pivotal roles during kidney embryogenesis. This research shows that glycolysis modulation affects nephron progenitor cells via Acetyl-CoA-modulated pathways, influencing both kidney development, and nephron endowment at birth.

## Introduction

Kidney function is directly proportional to nephron endowment at birth and inversely proportional to the development of hypertension in later life^1,2^. Nephron endowment at birth is governed by a balance between nephron progenitor cells (NPC) self-renewal and differentiation. Disturbances in NPC fate decisions cause low nephron endowment leading to hypertension and chronic kidney disease in adulthood^3,4^. The NPCs are a transient cell population spanning approximately 2 weeks in mice (from E11 to P4), and 30 weeks in humans (from week 5 to 35), and losses in the NPC pool cannot be rescued once the pool is exhausted^5–7^. Given the importance of NPC for proper kidney development, understanding the molecular cues that govern NPC fate decisions is paramount to preventing the developmental origin of adult kidney disease.

Adaptation in response to changes in the environment is a constant requirement for cells in all living organisms. For instance, cells are particularly sensitive to fluctuations in the levels of nutrients and  metabolites which may become rate-limiting in specific physiological and pathological conditions^8–11^. Recently, we and others have demonstrated  the importance of cell metabolites in normal kidney development^12–14^. Mechanistically, cell metabolites have been shown to connect environmental cues to protein function, chromatin modifications, and transcriptional regulation^13–23^.

Glycolysis is a conserved pathway that participates in several cellular processes and generates metabolites for posttranslational modifications that control protein structure and activity^17,24,25^. Glucose-derived metabolites such as ATP, acetyl-CoA, and NAD have been shown to regulate cell fate during development via epigenetic and non-epigenetic mechanisms^17,26,27^. However, the importance of glucose metabolism for proper kidney development is not fully understood.

The glycolytic flux inside cells is regulated at several steps, with phosphofructokinase-1 (PFK1) being the first rate-limiting enzyme in the pathway. PFK1 activity is controlled by the intracellular allosteric activator fructose 2,6-bisphosphate (F2,6BP), which is produced by 6-phosphofructo-2-kinase/fructose-2,6-bisphosphatase (PFKFB3)^28,29^. Glycolysis inhibition at the PFK1 level promotes NPC differentiation, at the expense of cap-mesenchyme (CM) depletion^13^. Changes in the intracellular levels of glycolysis-derived metabolites have been linked to cell fate switches in embryonic stem cells^26^ and to branching morphogenesis defects in the developing ureteric tree in the kidney^30^. Thus, in certain conditions (starvation, pharmacological block, gene mutations), these metabolites may become the determining factors that hamper developmental processes.

Here, proteomic and transcriptomic profiles of NPCs treated with the Wnt inducer CHIR99021 (CHIR) or the glycolysis inhibitor YN1 revealed that acetyl-CoA and Mevalonate metabolism are pivotal for NPC pool maintenance. Moreover, in-vivo reduction of glucose-originated acetyl-CoA in NPCs resulted in depletion of both CM and glomeruli counts at birth. This study identifies acetyl-CoA as a key metabolite for kidney development and sodium acetate as a potential exogenous substrate to promote kidney development.

## Results

### Glycolysis flux impacts NPC fate decisions

**Fig. 1:**
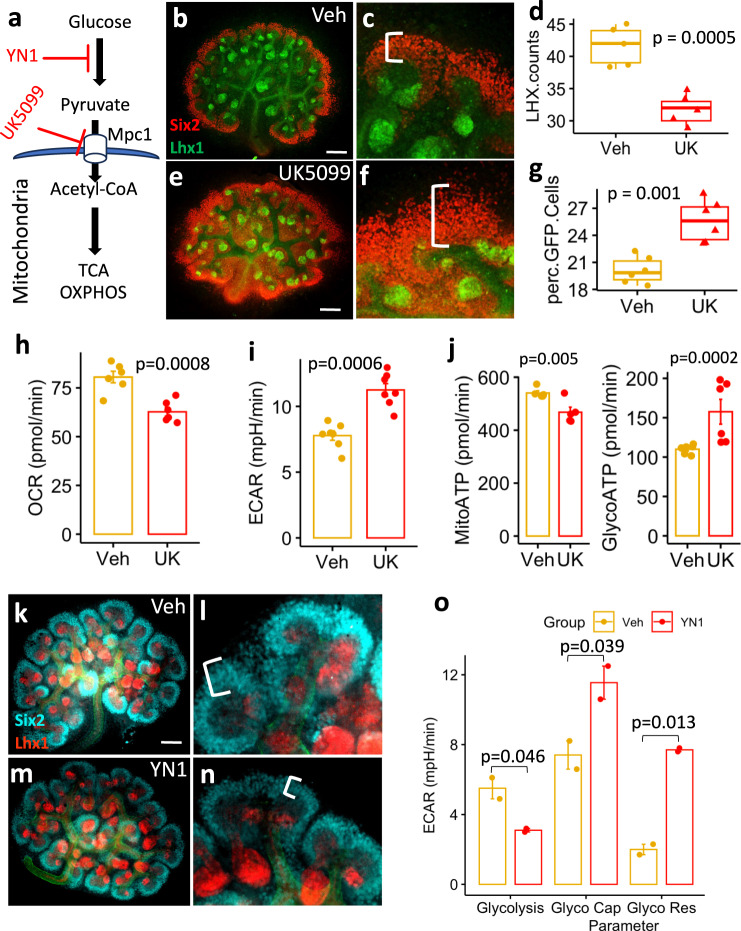
Glycolysis stimulation enhances the cap-mesenchyme. UK5099 is the inhibitor of mitochondrial pyruvate carrier (MPC1) that transports glucose-derived pyruvate into the mitochondria to feed the TCA cycle (**a**). Five pairs of E12.5 kidneys (*n* = 5 per condition) were cultured for 48 h in the presence of either vehicle (**b**, **c**) or 10 µM UK5099 (**e**, **f**) then fixed in 4% PFA and processed for immunostaining with anti-Six2 (red) or anti-Lhx1 (green). In parallel, E12.5 kidneys were treated as described above, fixed in 4% PFA, dissociated and FACS sorted based on GFP abundance from Six2GFPCre expression (*n* = 5 pairs of kidneys from 5 independent animals) or mounted and stained with anti-LHX1 antibody and the number of Lhx1 positive structures per kidney was counted (*n* = 6 pairs of kidneys from 5 independent animals). The number of Lhx1 positive structures per kidney and the percent of Six2 positive cells per kidney are plotted as boxplots, center lines denote medians, box limits indicate the 25th and 75th percentiles, and whiskers extend to the minimal and maximal values (**d**, **g**). The extracellular flux measurements were described in the methods section. Briefly, E13.5 NPCs (passage ≤ 3) were treated with 10 µM UK5099 or vehicle for 24 h, and standard parameters were measured as described in Methods. The oxygen consumption rate (OCR) was decreased (**h**, *n* = 2 independent experiments performed with 3 replicates) with a concomitant increase in extracellular acidification rate (ECAR) and glycolysis dependence (**i**, *n* = 2 independent experiments performed with at least 3 replicates) and a shift in ATP production towards glycolysis was observed (**j**, *n* = 2 independent experiments performed with 3 replicates) (data shown as the Mean ± SEM). E12.5 kidneys (*n* = 5 pairs of kidneys) were cultured for 24 h in the presence of either vehicle (**k**, **l**) or 20 µM YN1 (**m**, **n**) then fixed in 4% PFA and processed for immunostaining with anti-Six2 (cyan) or anti-Lhx1 (red) antibodies. E13.5 NPCs (passage ≤ 3) were treated with 20 µM YN1 for 24 h, and then the Glycolysis, Glycolytic capacity (Glyco cap), and Glycolytic reserve (Glyco Res) were measured with Seahorse respirometry (Mean ± SEM of 2 independent experiments, performed in triplicates) (**o**). Notice the white brackets in **c**, **f**, and **l**, *n* indicating the thickness of cap-mesenchyme. Scale bar = 100 µm. extracellular acidification rate (ECAR). Statistical comparisons between groups were performed with a two-sided Welch Two Sample t-test, *p*-values are reported in the figure). Source data are provided as a Source Data file.

To evaluate how glycolysis dependence affects kidney development, E12.5 mouse kidneys were treated with 10 µM UK5099, a pharmacological inhibitor of the Mitochondrial Pyruvate Carrier 1 (MPC1), for 48 h (Fig. 1a). UK5099 treatment increases cytosolic pyruvate and decreases mitochondrial acetyl-CoA formation^31^. Treatment with UK5099 resulted in an expansion of the CM and a reduction of Lhx1-positive nascent nephrons (Fig. 1b–g). As expected, cellular respirometry experiments showed that isolated E13.5 NPCs treated with 10 µM UK5099 for 24 h had decreased oxygen consumption rate (OCR) (Fig. 1h) and increased glycolysis dependence (Fig.1i). UK5099 treatment increased the glycolytic ATP production (Glyco ATP) and decreased the oxidative phosphorylation-driven ATP production (mito ATP) (Fig. 1j). Conversely, 24 h treatment of E12.5 kidneys with 20 µM YN1, a PFKFB3-specific glycolysis inhibitor, showed increased NPC differentiation at the expense of CM depletion (Fig. 1k–n and previously published^13^). Moreover, isolated E13.5 NPCs treated with 20 µM YN1 or vehicle for 24 h show decreased glycolysis with concomitant increased glycolytic capacity, glycolytic reserve, and non-glycolytic acidification (Fig. 1o). These results showed a strong association between glucose metabolism and NPC fate decision.

### Cell metabolism play roles in NPC fate

**Fig. 2:**
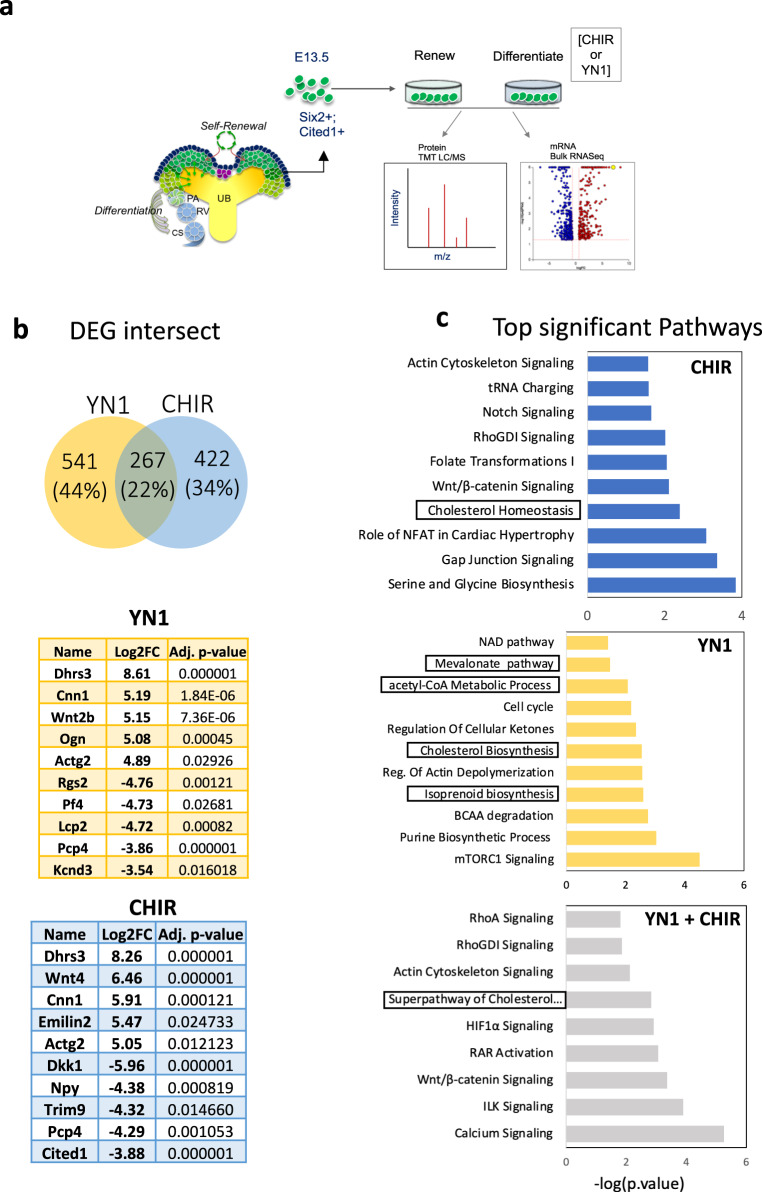
Transcriptome analysis of stimulated NPC shows metabolic pathways at the core of the differentiation process induced either by YN1 or CHIR. E13.5 NPC were isolated and cultured for 24 h with either Vehicle, YN1, or CHIR, and then subjected to either bulk RNA (*n* = 3 independent NPC preparations) or proteomic profiling (3 independent NPC preparations) (**a**). The bulk RNA analysis revealed common and treatment-specific differentially expressed genes (DEGs) in the NPCs treated with YN1 or CHIR (Venn diagram). The top-5 upregulated, and top-5 downregulated genes are shown (**b**). Bar plots show enriched terms further filtered for terms related to metabolism and developmental processes. Top terms were ordered by log10(p-value) obtained from the enrichment analysis with EnrichR (described in methods). Terms related to related to Mevalonate/cholesterol metabolism are highlighted in boxes (**c**). Source data are provided as a Source Data file.

To uncover potential molecular mechanisms by which glycolysis modulation impacts NPC fate decisions, we stimulated differentiation in E13.5 NPC with either YN1 or CHIR for 24 h and then performed transcriptome and proteomic analysis (Fig. 2a). Figure 2b shows the number of unique and shared genes between YN1 and CHIR. The transcriptome analysis revealed that CHIR treatment resulted in 689 DEGs compared to controls (311 upregulated and 378 downregulated genes) (Fig. 2b), impacting pathways related to cell metabolism (e.g.: t-RNA charging, Cholesterol synthesis, amino acid metabolism) and development (e.g.: WNT pathway, Rho GTPase, Notch signaling) (Fig. 2c). In comparison, YN1 treatment resulted in 808 DEGs (453 upregulated and 355 downregulated genes). The enriched pathways impacted by YN1 treatment were largely metabolic, as expected (Mevalonate pathway, acetyl-CoA metabolism, Purine metabolism) (Fig. 2b, c). Interestingly, 267 genes (22%) overlap between treatments with either drug (Fig. 2b, c, Supplementary Data 1 and 2). The cholesterol pathway (-Log10 *p*-value = 2.83), calcium signaling (–Log10 *p*-value = 5.26), and WNT signaling (–Log10 *p*-value = 3.36) scored among the top enriched pathways targeted by both drugs. Only DEG with a LogFC > |0.6 | , *p* < 0.05 were used for the enrichment analysis. Supplementary Data 1–4 contain the complete list of DEG and enriched GO terms.

YN1 treatment decreased the expression of genes related to glucose-to-acetyl-CoA metabolism. Several Glycolysis and TCA-cycle enzymes were downregulated by YN1 (Supplementary Fig.1a–c). Key enzymes related to citrate metabolism were significantly impacted, such as isocitrate dehydrogenase 1 and 2 (*Idh1*, LogFC = −0.6 and *Idh2*, LogFC = −0.8). Also, enzymes related to the conversion of citrate to acetyl-CoA formation in the cytosol (the citrate transporter *Slc25a1* (LogFC = −0.6), the ATP citrate lyase, *Acly* (logFC = −1.0) (Supplementary Fig.1b). Interestingly, Acyl-CoA Synthetase Short Chain Family Member 2, *Acss2*, an enzyme that converts acetate into acetyl-CoA was also downregulated (LogFC = −0.4, p-value > 0.05), but the p-value was not significant. The impact of YN1 treatment on histone deacetylases 1 and 2 expression was also not statistically significant (*Hdac1*, LogFC = −0.3, and *Hdac2*, LogFC = −0.2, *p*-value > 0.05), (Supplementary Fig.1b).

Furthermore, YN1 treatment decreased the expression of genes associated with naive NPC identity (*Osr1, Sall1, Six2, Cited1, Eya1, Gdnf*). Also, it promoted the upregulation of genes associated with NPC differentiation (*Wnt2b, Ncam1*) (Supplementary Fig. 1d) and phenocopied the effects of CHIR on NPC’s cell cycle (Supplementary Fig.1d). Treatment with YN1 led to the inhibition of cell-cycle progression genes (*Pcna, Birc5, Cks2, Ccnb1, Cdk4, Cdk1, and Gas1*) and upregulation of genes related to cell-cycle arrest (*Cdkn1a and Cdkn2a*) (Supplementary Fig. 1a and d).

We observed that YN1 treatment promoted upregulation of *Wnt2b* at the RNA level (LogFC = 5.15, p < 0.0001). However, several genes that constitute the canonical WNT machinery were downregulated by YN1 such as the WNT receptors (*Lrp5 and Fzd1*); intracellular machinery members (*Dvl1, Csnk1e, Axin1*); and effectors (*Tcf7*) (p < 0.05). YN1 treatment increased the expression of intracellular inhibitors of WNT signaling (*Cby1, Chd8, Nlk,* and *Smad3*). The expression of *Gsk3b* (LogFC = −0.4), a negative regulator of canonical WNT signaling, was decreased after 24 h of YN1 treatment, but it did not reach statistical significance (Supplementary Fig. 1d and 2b). These results suggest a connection between WNT signaling and glucose metabolism. However, the differentiation induced by YN1 treatment appears to be partially independent of WNT signaling, as demonstrated here and previously by our group^13^.

We conducted a Gene Enrichment analysis to gain insight into gene function and to identify biological pathways potentially impacted by both YN1 and CHIR. Changes in the Mevalonate/cholesterol pathway genes showed good concordance between the two treatments (YN1 and CHIR), pointing to a conserved involvement for this pathway in NPC fate determination (Fig. 2c). The canonical WNT signaling pathway (–Log10 *p*-value = 3.36), the actin cytoskeleton signaling (–Log10 *p*-value = 2.12), calcium signaling (–Log10 *p*-value = 5.26), and the RhoGDI/RhoA signaling (–Log10 *p*-value = 1.82) scored among the top hits for both drugs as well (Fig. 2b, c and Supplementary Data 3 and 4). As expected, YN1 treatment impacted metabolic pathways related to acetyl-CoA and cholesterol (Fig. 2c) preferentially.

Changes in gene expression in members of PI3K/MAPK pathway suggested a partial inhibition of this pathway after YN1 treatment (e.g.: PI3K catalytic unit and AKT1 were downregulated whereas PTEN, a negative regulator, was upregulated) (Supplementary Fig. 3a, b).

**Fig. 3:**
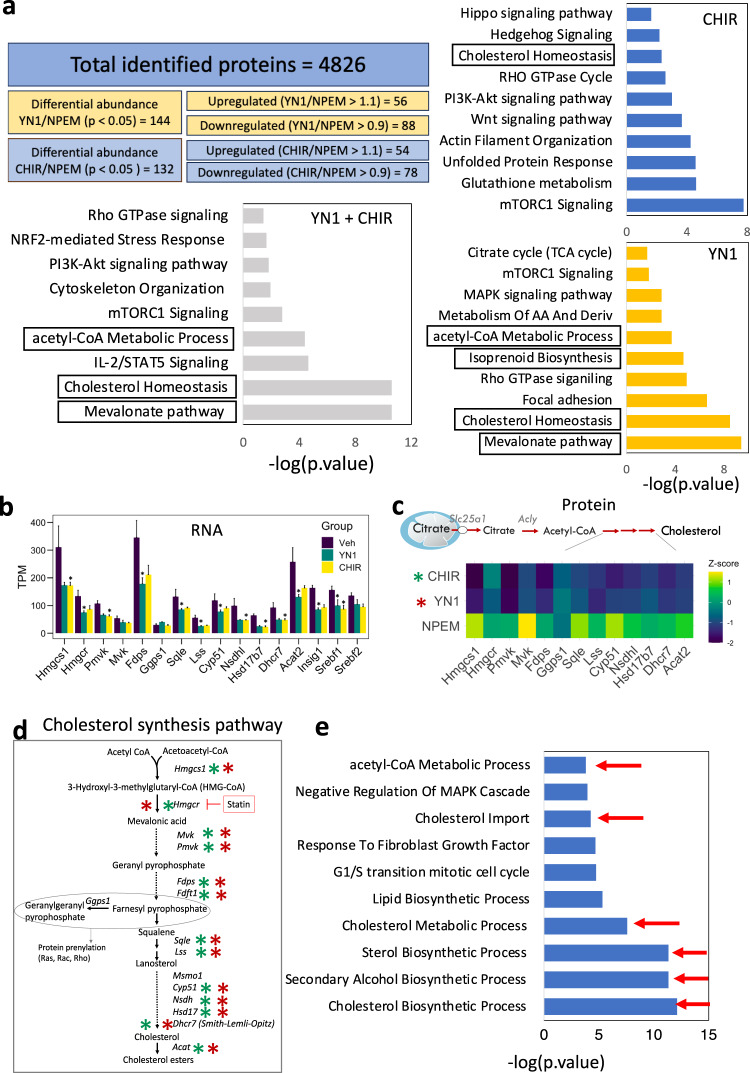
Proteomic analysis identifies metabolic and cytoskeleton-related pathways as common targets of YN1 and CHIR in NPC. Total identified proteins and filtering parameters used final up- and downregulated protein list per experimental condition. Differentially abundant proteins were used as input for enrichment analysis. Bar graphs show top-significant pathways impacted after each treatment and common pathways impacted by both drugs. Top terms were ordered by log10(p-value) obtained from the enrichment analysis with EnrichR (described in methods). Terms related to related to Mevalonate/cholesterol metabolism are highlighted in boxes (**a**). The transcripts encoding proteins from the cholesterol biosynthesis pathway were quantified and presented as the mean of transcript per million (TPM) +/- SEM, *n* = 3 independent NPC preparations (**b**). Heatmap generated to illustrate the downregulation of enzymes in the cholesterol biosynthesis pathways after treatment with YN1 or CHIR, at the protein level. Data are presented as the z-score obtained from 3 independent NPC preparations (**c**). The schematics of the cholesterol biosynthesis pathway are provided, with a significant decrease in pathway enzymes indicated by a cyan (YN1) or red (CHIR) asterisk (**d**). Top terms from GO analysis with downregulated genes as input after YN1 treatment, scores are shown as log10(p-value) (**e**). Source data are provided as a Source Data file.

Proteomic profiling of stimulated NPCs showed a high concordance with the transcriptomic profiles and resulted in 4826 proteins shared between CHIR and YN1 treatments. Analysis of significant differential protein abundance ratios from NPC induced either with YN1 (YN1/NPEM = 144 proteins *p* < 0.05) or CHIR (CHIR/NPEM = 132 proteins *p* < 0.05) show that several top pathways overlap between treatments (Fig. 3a). The cholesterol pathway was a top hit impacted at both the transcript and protein levels (Fig. 3a–e). The treatment either with YN1 or CHIR affected the mTOR pathway, which has been associated with NPC lifespan^14^. Changes in the mRNA of genes involved in amino acid biosynthesis and tRNA charging pathways further confirmed these observations (Figs. 2 and 3).

### YN1 does not promote uncontrolled stress responses in NPCs

Pharmacological treatments may induce uncontrolled stress responses and prompt cells to differentiate^32–35^. For this reason, we investigated whether YN1 or CHIR treatment impacted the expression of oxidative stress response genes. We measured the fold change of eight genes related to NRF2-mediated oxidative stress response (Nf2l2, Nqo1, Hmox1, Gclc, Gclm, Cat, Sod1, Sod2)^36^, five Glutathione peroxidase genes expressed in the developing kidney (GPX1,−3,−4,−7,−8)^37^ and five target genes activated during oxidative stress response in cells (Cdkn1b, Trp53inp1, Hif1a, Id2, Foxo4)^30^. The expression of these genes was compared between control and YN1 cohorts or control and CHIR cohorts. The results were considered significant if they showed an absolute LogFC difference greater or equal to |0.5| with a p-value < 0.05. Genes in the NRF2 pathway were either downregulated or did not show significant changes. Additionally, there were no significant differences in the expression of the other genes tested across conditions (as shown in Supplementary Fig. 4a, b). Although not statistically significant, we noticed an increase in LogFC values for a few genes (Gpx3, Gpx7, Gpx8, Trp53inp1) after YN1 treatment (Supplementary Fig. 4a).

It has been shown that uncontrolled stress response disturbs cell homeostasis resulting in changes in protein synthesis^38,39^. Therefore, we evaluated whether the cellular protein synthesis machinery was impacted by treatment with either YN1 or CHIR. Our results showed no statistically significant differences in the transcription rates of ribosomal protein-coding genes after treatment with either drug (as shown in Supplementary Fig. 4c, d, f, adj_*p*-value < 0.05). More importantly, there were no differences in the total abundance of ribosomal proteins observed with proteomic experiments after treatment with YN1. Nonetheless, the proteomics analysis showed increased ribosomal protein abundance after Wnt signaling stimulation with CHIR (as shown in Supplementary Fig. 4e, Kruskal-Wallis test). Given that Wnt stimulation is a potent signal for differentiation^6,40,41^, it was anticipated.

### Reduction in cholesterol synthesis enables NPC differentiation

Multiple enzymes in the cholesterol synthesis pathway were significantly downregulated by YN1 or CHIR treatment, at mRNA (Fig. 3b) and protein levels (Fig. 3c and Table 1). The impacted enzymes are epistatically organized in Fig. 3d. We subset the downregulated genes after YN1 treatment and ran another GO analysis. This deeper analysis confirmed terms related to imbalances of cholesterol metabolism scored as top significant hits (Fig. 3e, red arrows). Interestingly, the steroid regulatory element binding factors, common upstream regulators for multiple enzymes in this pathway, were also downregulated. Both Srebf1 (LogFC = −0.76, avg exp = 6.97, *p* < 0.05) and Srebf2 (LogFC = −0.47, avg exp = 6.92, *p* = 0.08) transcripts were reduced following YN1 treatment, but statistical significance was only achieved with Srebf1. This data suggested that the maintenance of the NPCs requires cholesterol synthesis, and disruption of the cholesterol synthesis machinery prompts them to differentiate.

**Table 1:** Differentially abundant proteins in the Super-pathway of Cholesterol Biosynthesis network

Protein name	YN1/Ctrl	CHIR/Ctrl
Acat1	−1.22619	−0.1422
Lbr	−1.11595	−1.03956
Hadhb	−1.0669	−1.09432
Hadha	−1.0977	−1.07973
Hmgcr	−1.34085	−1.27025
Hsd17b7	−1.47669	−1.47494
Sqle	−1.44067	−1.53392
Msmo1	−1.54533	−1.46496
Fdft1	−1.51828	−1.56956
Lss	−1.58772	−1.52997
Tm7sf2	−1.60068	−1.54865
Nsdhl	−1.58253	−1.67584
Dhcr7	−1.63414	−1.69493
Fdps	−1.58861	−1.78847
Acat2	−1.74065	−1.8132
Cyp51a1	−1.78618	−1.87085
Mvk	−1.84842	−1.90875
Pmvk	−1.89759	−2.02919
Idi1	−2.00878	−2.01207
Mvd	−2.03588	−2.03867
Hmgcs1	−1.88482	−2.29426

The proteomic data was filtered to include proteins associated with the Mevalonate/cholesterol pathway. Protein abundances are represented as the ratio of the grouped abundance in the treatment groups to the grouped abundance in the control media (YN1/Ctrl and CHIR/Ctrl).

**Fig. 4:**
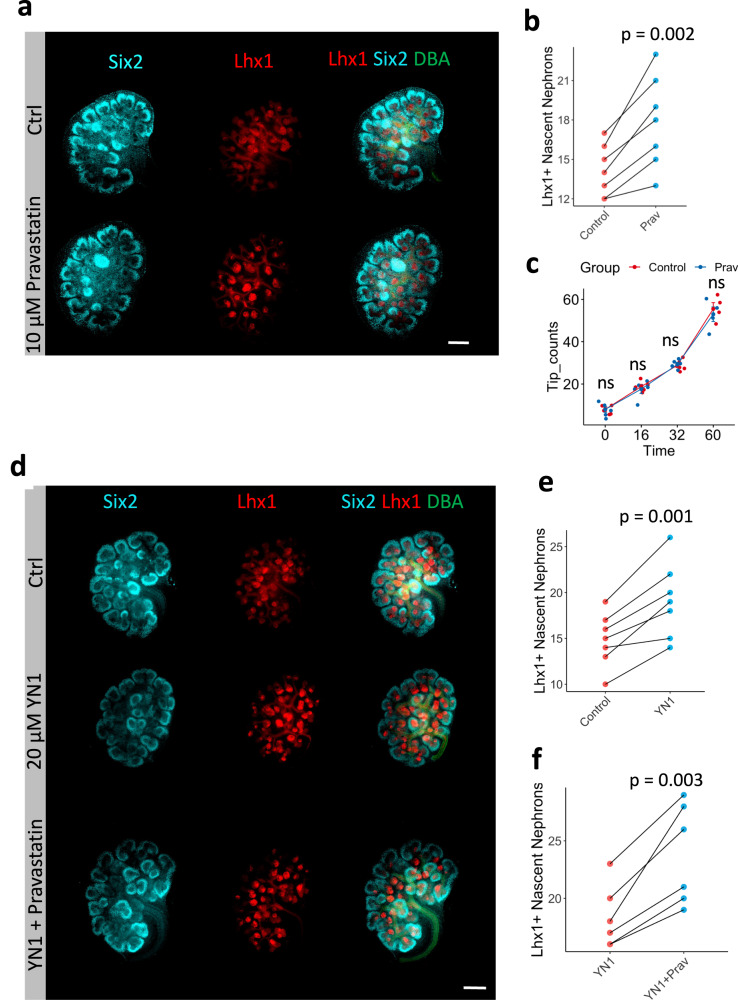
Inhibition of the cholesterol synthesis pathway phenocopy YN1 treatment and drives NPC differentiation. Six2 (NPC) Lhx1 (nascent nephron), and DBA (UB) immunostaining in E12.5 kidneys after 24 h incubation in the presence of 10 µM Pravastatin (Prav) or vehicle (ctrl) (**a**). Quantification of the number of LhX1+ structures, *n* = 7 kidney pairs from 7 independent embryos (**b**). Quantification of UB tip branching up to 60 h incubation period in the presence of 10 µM pravastatin, *n* = 5 pairs of kidneys per time point, Data presented as mean +/- SEM and individual dots are depicted (**c**). E12.5 + 24 h treatment with control (vehicle), 20 µM YN1 or YN1 + 10 µM pravastatin treatment (**d**). Quantification of nascent nephron after YN1, *n* = 7 pairs of kidneys from independent embryos (**e**) or Prav + YN1 treatment, *n* = 6 pairs of kidneys from independent embryos (**f**). Six2 (Cyan); Lhx1 (Red), DBA (Green). Scale bar 250 µm. In all experiments, each pair of kidneys was separated and designated to receive vehicle or treatment randomly. After treatment, the pairs were compared with the two-sided T-test for paired samples, and p-values were shown. Source data are provided as a Source Data file.

To confirm that cholesterol metabolism is important for NPC pool maintenance, we cultured E12.5 embryos for 24 h in the presence or absence of 10 µM of Pravastatin, an inhibitor of HMG-CoA reductase (see Fig. 3d). Inhibition of cholesterol synthesis increased the number of Lhx1 positive structures (Fig. 4a, b), without affecting UB tip number counts over time (Fig. 4c).

Interestingly, co-treatment (Pravastatin + YN1) increased differentiation above the level of either drug alone (Fig. 4d–f). Thus, a partial block of glycolysis may have effects on nephrogenesis that are either independent of cholesterol biosynthesis or have impacted an upstream metabolite also required for cholesterol synthesis.

### Acetyl-CoA links glycolysis inhibition to cholesterol synthesis

**Fig. 5:**
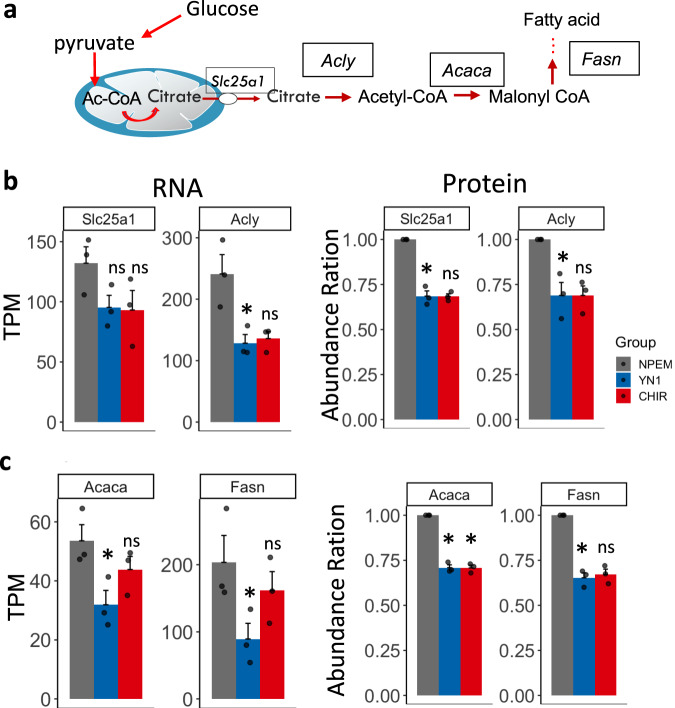
Acetyl-CoA is a key metabolite at the crossroad between cholesterol, and fatty acid synthesis. Glycolysis-generated acetyl-CoA metabolic route (**a**). Transcript (TPM) and protein/peptides (Abundance) quantification of key acetyl-CoA generating enzymes after treatment with either Nephron progenitor expansion media (NPEM) (grey bar, used as a control media), YN1 (Blue bar) or CHIR (red bar) (**b**). Quantification of key transcript and proteins in fatty acid pathway after treatment with either YN1 or CHIR (**c**). Data presented as the mean of transcript per million (TPM) for RNA or abundance ratio for proteomic +/- SEM, *n* = 3 independent NPC preparations. ATP-citrate lyase (Acly), the mitochondrial citrate transporter (Slc25a1), the Fatty acid synthase (Fasn), and acetyl-CoA carboxylase alpha (Acaca). Multiple samples were compared with the Kruskal-Wallis test, followed by Holm’s posthoc test, *posthoc *p*-value <or = 0.05 was considered significant, ns = not significant. Source data are provided as a Source Data file.

Acetyl-CoA is an end-product of glycolysis and a precursor for cholesterol synthesis. Thus, it is a key metabolite linking glycolysis inhibition to cholesterol biosynthesis (Fig. 5a).

The cytosolic pool of acetyl-CoA is largely dependent on mitochondrial citrate shuttle and metabolism^42,43^. *Slc25a1* encodes the mitochondrial membrane transporter that shuttles citrate to the cytosol, whereas ATP-Citrate lyase (Acly) is a cytosolic/nuclear enzyme that converts mitochondria-derived citrate to acetyl-CoA (Fig. 5a). Treatment either with YN1 or CHIR reduces the expression of *Slc25a1* and *Acly* at transcript levels but was statistically significant only for *Acly* expression after YN1 treatment when the TPM was evaluated (Fig. 5b). However, we found significant changes when the LogFC between Ctrl and YN1 was evaluated (*Acly* LogFC = −0.54, avg.exp= 6.8, *p*-value < 0.05, Supplementary Fig. 1). The reduction of Slc25a1 and Acly was also confirmed at protein levels and found to be statistically significant for both, after YN1 treatment (Fig. 5b).

Both YN1 and CHIR promoted the downregulation of enzymes in the fatty acid synthesis pathway. Fatty acid synthase (*Fasn*) and acetyl-CoA carboxylase alpha (*Acaca*) were downregulated, at the RNA and protein level after YN1 treatment (Fig. 5c). After CHIR treatment, although we observed a reduction in both Fasn and Acaca, statistical significance was only found with Acaca at protein levels (Fig. 5c).

### Sodium Acetate supplementation favors NPC pool maintenance

**Fig. 6:**
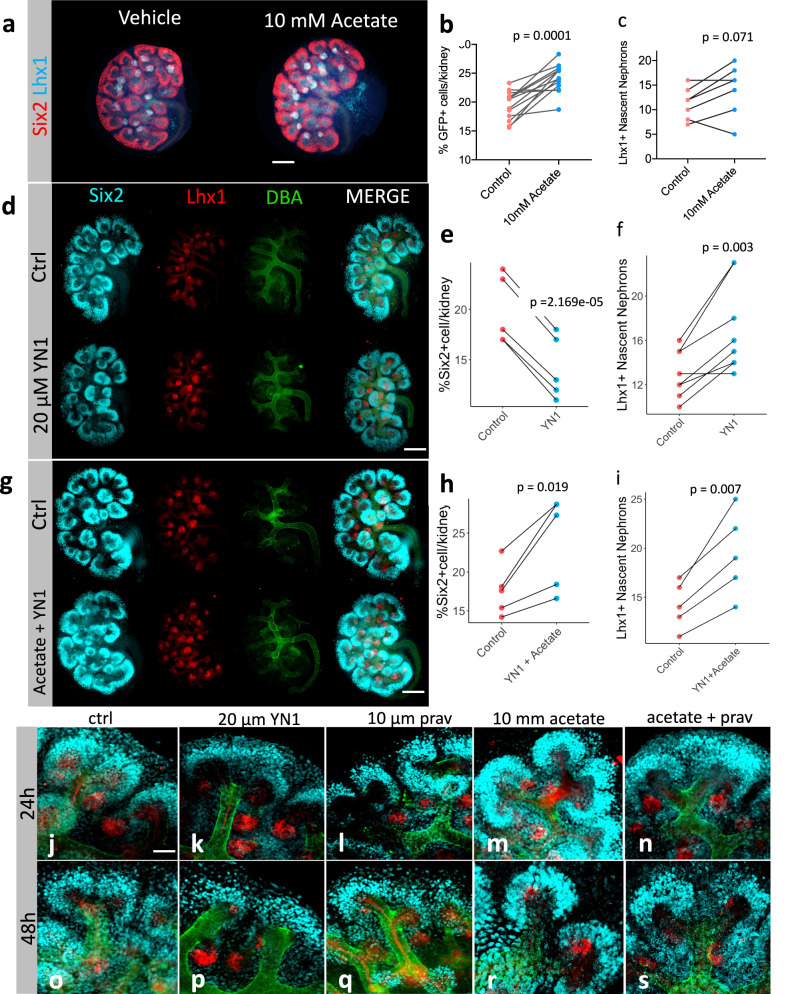
Sodium acetate boosts CM development and prevents drug-induced depletion Six2^+^ population. Immunostaining of E12.5 embryonic kidneys after 24 h treatment in the presence of vehicle or 10 mM sodium acetate (**a**). Quantification of GFP^+^- cells by FACS in both control and sodium acetated treated cells, *n* = 13 pairs of kidneys from independent embryos (**b**). Quantification of Lhx1+ nascent nephrons in immunostaining of E12.5 embryonic kidneys after 24 h treatment in the presence of vehicle or 10 mM sodium acetate, *n* = 6 pairs of kidneys from independent embryos (**c**). Immunostaining of E12.5 embryonic kidneys after 24 h treatment in the presence of 20 µM YN1 or vehicle (**d**). Quantification of Six2^+^ cells, *n* = 6 pairs of kidneys from independent embryos (**e**), or Lhx1-nascent nephrons, *n* = 8 pairs of kidneys from independent embryos (**f**). E12.5 embryonic kidneys after 24 h treatment in the presence of a combination of 20 µM YN1 + 10 mM sodium acetate or vehicle (**g**). Quantification of Six2^+^ cells, *n* = 5 pairs of kidneys from independent embryos (**h**), or Lhx1^+^-nascent nephrons, *n* = 5 pairs of kidneys from independent embryos (**i**). Prolonged culture of E12.5 kidneys in the presence of vehicle (**j**, 24 h, and o 48 h); YN1 (**k**, 24 h and p 48 h); Pravastatin (Prav) (**l**, 24 h, and q 48 h); Sodium acetate (**m**, 24 h, and r 48 h); sodium acetate + Pravastatin (**n**, 24 h and s 48 h). All groups with 48 h cultures comprised 5 pairs of kidneys from independent embryos/per group. Scale bar in **a**, **d**, and G = 250 µm. Scale bar in **j**–**s** = 10 µm. In all experiments, each pair of kidneys was separated and designated to receive vehicle or treatment randomly. After treatment, the pairs were compared with the two-sided T-test for paired samples, and p-values were shown. Source data are provided as a Source Data file.

Since the cytosolic acetyl-CoA-producing machinery was downregulated by glycolysis inhibition, we hypothesized that restoring the intracellular pool of acetyl-CoA would favor NPC pool maintenance. Acetate is a 2-carbon molecule that shuttles between cells and can be easily converted to acetyl-CoA once inside^44^. E12.5 mouse kidneys cultured for 24 h in the presence of 10 mM sodium acetate showed a larger CM compared to controls (Fig. 6a). Sodium acetate effects on developing kidneys were concentration-dependent (Supplementary Fig. 5). We dissected E12.5 kidneys from embryos bearing a copy of the Six2^GFP-Cre^ transgene^45^ and treated them either with vehicle or 10 mM sodium acetate for 24 h, then the Six2 positive cells were counted by flow cytometry. Sodium acetate treatment increased the percentage of GFP-positive cells per kidney (Fig. 6b). This finding correlated with the higher Six2 protein signal after sodium acetate supplementation (Supplementary Fig. 5). The treatment did not affect the formation of Lhx1-positive nascent nephrons (Fig. 6c). Indicating that sodium acetate supplementation did not hinder cell differentiation.

Co-incubation of E12.5 kidneys with 20 µM YN1 and 10 mM sodium acetate abrogated the YN1-driven depletion of CM (compare Fig. 6d, e, and g, h) without impairing differentiation of Lhx1 positive structures (compare Fig. 6f and i). The impact of sodium acetate supplementation on YN1-treated kidneys was dosage-dependent (Supplementary Fig. 6). Incubation of E12.5 kidneys with the combination of 20 µM YN1 + 1 mM sodium acetate was sufficient to prevent YN1-driven depletion of cap-mesenchyme. When compared to normal kidneys, E12.5 kidneys treated with 20 µM YN1 in the presence of 10 mM sodium acetate showed larger CM and higher Six2 fluorescence intensity (Supplementary Fig. 6). These ex-vivo experiments indicated that the effects of YN1 on nephrogenesis were largely dependent on the acetyl-CoA levels. In general, sodium acetate treatment does not appear to favor a particular fate rather it potentiates kidney development favoring NPC pool maintenance and the formation of nascent nephrons.

Next, we investigated whether prolonged exposure to metabolic perturbation impacted kidney development. Five pairs of E12.5 kidneys per condition were cultured for 24 or 48 h in the presence of a vehicle, or drug. Compared to the control (Fig. 6j 24 h and Fig. 6o 48 h), treatment with YN1 (Fig. 6k 24 h and Fig. 6p 48 h) and Pravastatin (Fig. 6l 24 h and Fig. 6q 48 h) resulted in a decrease in cap-mesenchyme, with YN1 having a more significant effect than Pravastatin. Sodium acetate treatment, on the other hand, led to an enlargement of the CM (Fig. 6m 24 h and Fig. 6r 48 h). Pravastatin did not prevent sodium acetate effects on CM or the formation of nascent nephrons (Fig. 6n 24 h and Fig. 6s 48 h). The effects of the drugs were more pronounced at 48 h, and interestingly, even after 72 h of incubation, acetate supplementation was able to promote kidney development (Supplementary Fig. 7a–d). Furthermore, we measured the intracellular formation of acetyl-CoA after 24 h acetate supplementation and it was increased indicating that sodium acetate supplementation was sufficient to increase intracellular acetyl-CoA formation (Supplementary Fig. 7e).

### Removal of *Acly* in Six2-expressing cells depleted NPC pool

**Fig. 7:**
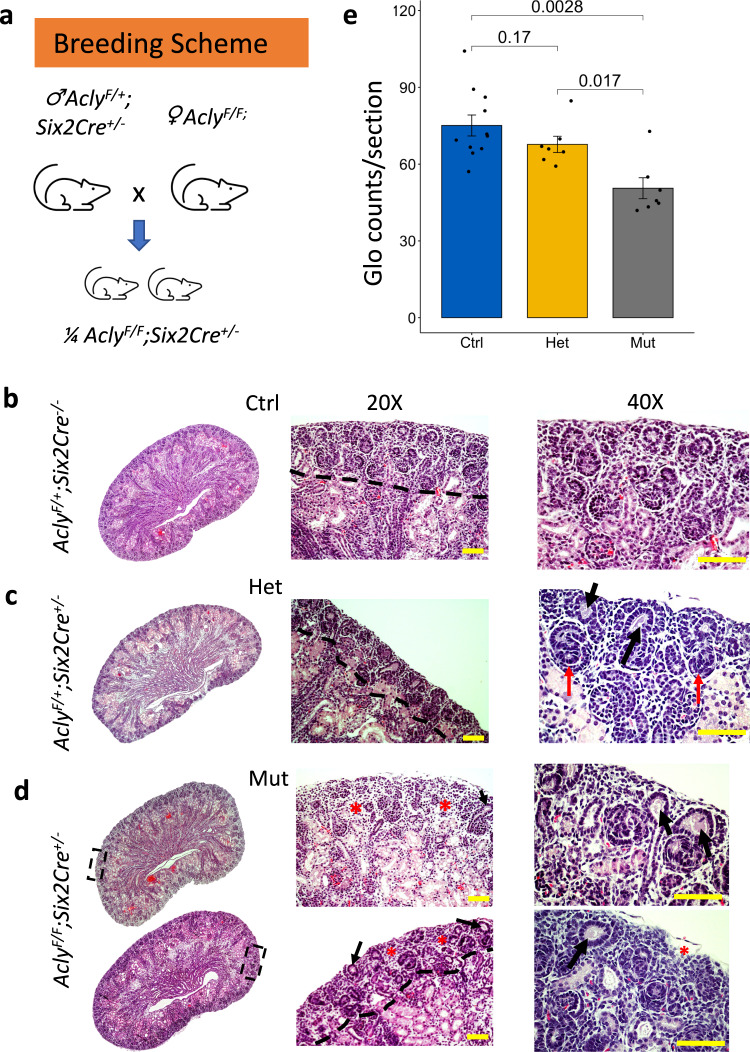
Morphological characterization of mutant kidneys lacking *Acly* expression in the CM at P0. Breeding strategy to generate embryonic kidneys lacking *Acly* expression in the CM cells (**a**). H&E-stained control kidney (**b**), heterozygous kidneys (**c**), and mutant kidneys (**d**). The nephrogenic zone is marked with a dashed line at 20X. Detailed structures can be observed at 40X magnification. The nephrogenic zone is narrower in heterozygous and mutant kidneys. Detailed structures observed at 40X magnification revealed dilations at UB ampullae (black arrow) and the presence of some nascent nephrons (red arrows), CM around dilated UB ampullae appears depleted and almost absent in some areas of mutant embryos. Also, notice expansion of interstitial tissue (red asterisk) (**d**). Glomeruli counts at P0 in control (blue, *n* = 11 independent kidneys), heterozygous kidneys (gold, *n* = 7 independent kidneys), and mutant embryos (grey, *n* = 7 independent kidneys) (**e**), scale bar 100 µm. A Kruskal-Wallis test for multiple comparisons was used to compare groups and *p*-values are shown. Source data are provided as a Source Data file.

The Acly reaction is the major source of acetyl-CoA in the cytosol and nuclear environments^42,46^. Thus, we hypothesize that the removal of *Acly* in NPC would impact nephrogenesis in-vivo. We took advantage of a transgene expressing Cre-recombinase under the control of the Six2 promoter to remove *Acly* from the Six2 expressing NPC population (*Six2Cre::Acly*^*-/-*^) in the developing kidney (Fig. 7a). P0 mutant kidneys had several morphological abnormalities such as poorly developed nephrogenic zone (Fig. 7b, d, dashed lines), dilation of UB ampullae (Fig. 7d, black arrows, 20X, and 40X), and increased stromal mass surrounding scarce nephrogenic niches (Fig. 7e, red asterisk, 20X, and 40X). Often, we observed depleted CM around these dilated UBs (Fig. 7d, 20X, and 40X). Heterozygous animals appeared to have a slightly reduced nephrogenic zone with variations observed between embryos (Fig. 7c). We observed the mutant phenotype varied, and more impacted embryos had regions with fewer or no nascent nephrons (Fig. 7d, top mutant kidney). However, in other mutants, we observed the presence of some nascent nephrons though nephrogenesis was defective (Fig. 7d, bottom right kidney). H&E staining from whole kidney sections confirmed the variability of the phenotype (Supplementary Fig. 8). Nonetheless, mutant embryos presented significantly narrower nephrogenic zones and fewer nascent nephrons. The efficiency and specificity of the Six2GFP-Cre-mediated conditional deletion of *Acly* was confirmed by immunofluorescence (Supplementary Fig. 9).

At P0, control (*n* = 12 embryos), heterozygous (*n* = 7 embryos), and mutant kidneys (*n* = 7 embryos) from 3 separate litters were sectioned and the number of glomeruli per section was counted at the mid-coronal level. We found a reduction of roughly 30% glomeruli counts in the mutant embryos compared to controls (75 ± 13 and 49 ± 10 glomeruli/section, control, and mutant, respectively) (Fig. 7e). No significant differences were observed between control and heterozygous embryos. Though a reduction in the number of glomeruli was noticeable (67 ± 8) (Fig. 7e). Heterozygous kidneys have a significantly higher number of glomeruli than mutants (Hets=67 ± 8 vs Mut = 49 ± 10 glomeruli/section, Kruskal-Wallis test, *p* < 0.05).

**Fig. 8:**
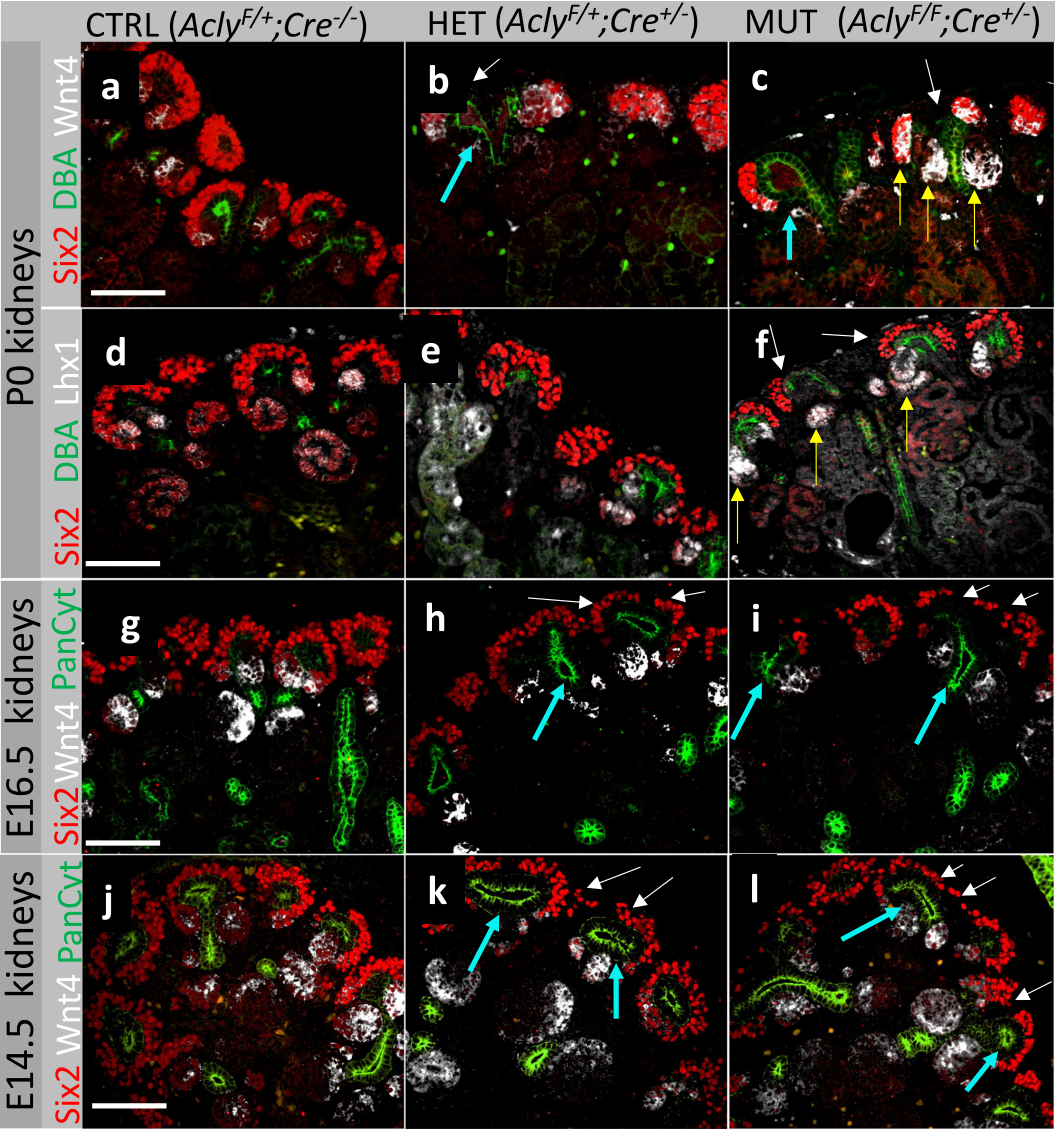
Molecular characterization of developmental defects resulting from ablation of Acly in Six2^+^ cells. Visualization of nephrogenic niches of control kidneys (**a**, **d**), heterozygous (**b**, **e**), and mutant kidneys (**c**, **f**) at P0. E16.5 kidneys are displayed as control kidneys (**g**), heterozygous (**h**), and mutant kidneys (**i**). E14.5 kidneys are shown as control kidneys (**j**), heterozygous (**k**), and mutant kidneys (**l**) Cyan arrows point to dilated UB ampullae; white arrows show areas with depleted cap-mesenchyme; yellow arrows show expansion of Wnt4 towards the cap-mesenchyme. Five embryonic kidneys per genotype per stage were used in this experiment. Embryos from at least two distinct litters per stage and genotype were sectioned and stained. Scale bar 100 µm.

The CM of P0 WT embryos showed Six2^+^ cells forming a crescent shape around the UB and Wnt4 and Lhx1 expression was detected in the PTA and RV structures (Fig. 8a, d, S10a–e, S10p–t). The Six2^+^ cell population was affected in heterozygotes but to a lesser extent. Heterozygotes showed a dilation of the UB ampullae and expansion of Wnt4, but not Lhx1, towards the CM (Fig. 8b, e). *Six2Cre::Acly*^*-/-*^ mutant embryos showed an abnormal dilation of UB ampullae and a reduction of Six2^+^ cells. The mutant kidneys also had an expanded Wnt4 expression and stronger Lhx1 staining (Fig. 8c, f, and Supplementary Fig. 10f-o, u-y). These results suggested that *Acly* depletion in Six2^+^ cells led to ectopic activation of the *Wnt4 locus*, and it may have pushed naïve NPC into differentiation rather than self-renewal. The effects of the removal of *Acly* in Six2^+^ cells were detected at early time points. For instance, dilations of the UB ampullae were evident at E16.5, and present but less prominent at E14.5 in both heterozygotes and mutant kidneys (Fig. 8g–l, yellow arrows). The CM was depleted at E14.5 and E16.5 in mutant kidneys and in some heterozygous kidneys (Fig. 8g–l, white arrows).

**Fig. 9:**
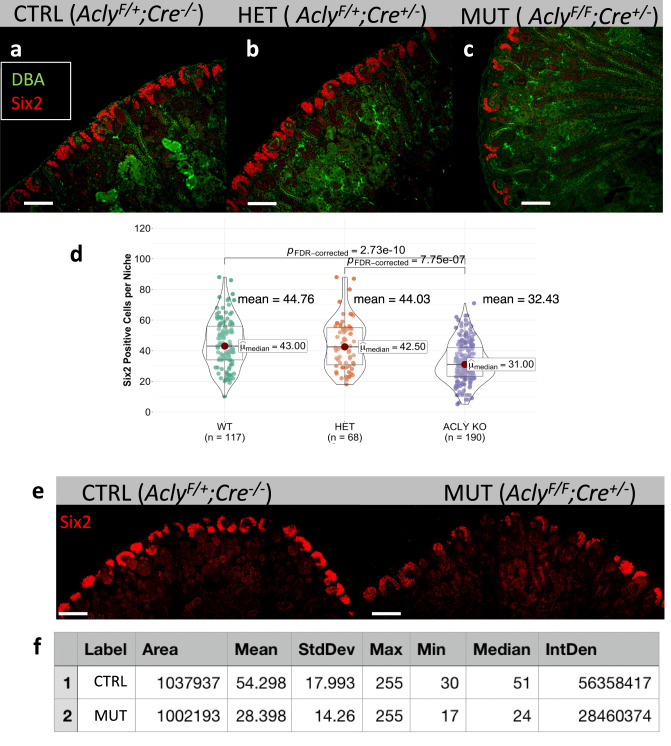
Deletion of *Acly* in Six2+ cells resulted in depletion of CM and reduction in Six2 protein abundance at P0. P0 control (*Acly*^*F/*^*;Cre*^*-/-*^), heterozygous (*Acly*^*F/+*^*;Cre*^*+/-*^), and mutant kidney (*Acly*^*F/F*^*;Cre*^*+/-*^), stained with DBA (green), Six2 (Red) merged **a**–**c** Quantification of Six2 positive cells per niche was performed by counting all Six2+ cells in each niche. Notice a statistically significant reduction of Six2^+^cells detected in mutant kidneys compared to control and heterozygous kidneys, number of niches counted is depicted, center lines with maroon dot denote medians, mean values are placed in the figure, box limits indicate the 25th and 75th percentiles, and whiskers extend to the minimal and maximal values (**d**). Selection of Six2 immunofluorescence signal of control and mutant kidneys within a similar area and the number of niches, roughly 20 (**e**). Quantification of fluorescence intensity shown in E, area, mean intensity, min, max values, and median are shown (**f**). Kruskal-Wallis test was performed across groups, global *p*-value = 5.70e^−12^, *n* = 375 observations. The pairwise comparison was performed with the Dunn test, significant *p*-value < 0.05. FDR-corrected values are presented in the figure. Scale bar 200 µm. Source data are provided as a Source Data file.

Finally, to assess the extent of CM depletion after *Acly* ablation in NPC, we counted the Six2^+^ cells, per niche, in P0 control (*Acly*^*F/+*^*;Cre*^*-/-*^), heterozygotes (*Acly*^*F/+*^*;Cre*^*+/-*^), and mutant embryos (*Acly*^*F/F*^*;Cre*^*+/-*^) (Fig. 9A–C). Compared to wild-type kidneys (WT, mean = 45 Six2^+^cells/niche), Acly mutants presented significantly lower numbers of Six2^+^ cells (Acly KO, mean = 32 cells Six2^+^cells/niche, *p* > 0.001). However, WT counts were not significantly different from heterozygotes kidneys (HET, mean = 44 cells Six2^+^cells/niche). As expected, heterozygous kidneys presented significantly higher numbers of Six2^+^ cells compared to *Acly* mutants (*p* > 0.001) (Fig. 9d). To confirm these results, we quantified the fluorescence intensity of Six2^+^ cells in a large portion of the P0 kidney (roughly ~20 niches). The control kidneys had nearly twice as much fluorescence as mutants within the same region size (Control, mean intensity = 54.298 and MUT-Acly KO, Mean intensity = 28.398) (Fig. 9f). In addition, we found that the minimal and the mean fluorescence intensity was reduced by a factor of two in mutants, confirming the significant reduction in Six2 protein levels after *Acly* removal (Fig. 9f).

These results phenocopied YN1 treatment by decreasing Six2 protein levels (Figs. 4 and 6). Conversely, sodium acetate treatment increased Six2 protein levels in ex-vivo cultures (Fig. 6). Therefore, we hypothesized that sodium acetate supplementation could rescue the *Acly* mutant phenotype. To test this hypothesis we incubated E12.5 (CRL, MUT) kidneys for 24 h with 10 mM sodium acetate or vehicle, and verified that sodium acetate was capable of increasing cap-mesenchyme size in E12.5 Six2Cre::Acly^-/-^ mutant kidneys (Supplementary Fig. 11). Taken together, these experiments indicated Six2 levels positively correlate with acetyl-CoA availability in NPC.

## Discussion

A major finding of our study is that glycolysis-derived acetyl-CoA is a key metabolite for NPC pool maintenance and nephron mass determination at birth. This conclusion is supported by the following observations: 1) The NPC pool maintenance is directly proportional to the rate of glycolysis which is a major determinant of acetyl-CoA formation; 2) Acetyl-CoA is the main substrate for the Mevalonate pathway and members of the Mevalonate/cholesterol pathway are downregulated during NPC differentiation by both WNT stimulation or glycolysis inhibition; 3) treatment with a Mevalonate/cholesterol pathway inhibitor (Pravastatin), increases differentiation rates similar to glycolysis inhibition; 4) Sodium acetate supplementation increases NPC pool; and 5) Six2Cre-mediated removal of *Acly* leads to NPC pool depletion in vivo. Our findings shed light on the impact of glucose metabolism in kidney development and corroborate our group’s previous observation that glycolysis inhibition triggers NPC differentiation^13^. In support of these arguments, a recent publication reported that pyruvate, the end-product of glycolysis, plays a role in proper nephrogenesis^12,13^ and ureteric bud branching morphogenesis^30^. This mechanism appeared to be conserved in intestinal stem cells in which stimulation vs inhibition of glycolysis had opposite effects on proliferation and differentiation^47^.

Another key finding of this study is that Mevalonate/cholesterol biosynthesis is required for proper nephrogenesis. This conclusion stems from observations that Pravastatin treatment increases NPC differentiation. Moreover, both glycolysis inhibition and WNT stimulation are accompanied by downregulation of the Mevalonate/cholesterol synthesis machinery during NPC differentiation (Figs. 2–4). Although the underlying mechanisms were not directly investigated, acetyl-CoA is the main substrate of the Mevalonate/Cholesterol pathway. The mevalonate pathway branches into cholesterol and isoprenoid synthesis and is essential for all animal life. Cholesterol is required to maintain membrane integrity, fluidity, and metabolic activity of cells^48,49^. The isoprenoid pathway is required for several proteins to anchor to the plasma membrane to carry out their functions, such as Ras/Rho small GTPases^50,51^. The Ras/Rho small GTPases pathway was downregulated after YN1 treatment (Figs. 2 and 3). Thus, perturbations in glucose metabolism had pleiotropic effects on kidney development culminating in reduced proliferation and increased differentiation leading to NPC pool depletion (Supplementary Fig. 1). Interestingly, proper regulation of metabolites from the Mevalonate pathway has been shown to be pivotal for heart development in mice^52^, zebrafish^53^^,^ and Drosophila^54^. These findings suggest the mevalonate pathway, may play a conserved role in organ development across different *phyla* and organ systems. Because the synthesis of mevalonate is dependent on acetyl-CoA, it is possible that acetyl-CoA may also play a conserved role in organ development in different tissues and organisms.

Interestingly, Pravastatin treatment increased NPC differentiation even in the presence of excess sodium acetate. Thus, the Mevalonate pathway flux is important for NPC maintenance. However, these experiments also suggested that the effects of acetate/acetyl-CoA on NPC self-renewal appeared to be partially independent of the mevalonate pathway. It is possible that sodium acetate increases the NPC pool by targeting the proliferating NPC subpopulation, and once the cell is primed to differentiate and the genes in the mevalonate pathway are downregulated, the supplementation of sodium acetate cannot prevent differentiation. In the future, single-cell RNA sequencing experiments will help to sort and analyze the effects of acetate supplementation on specific subpopulations of NPC.

Recently, acetyl-CoA, compartmentalized and generated from pyruvate-derived acetate in the nuclei of endothelial cells, was shown to control endothelial-to-mesenchymal transition in the endothelium during the development of chronic vascular disease^55^. Altogether, these results inarguably reveal the importance of compartmentalized acetyl-CoA metabolism for cell biology during development and disease-related processes.

The WNT signaling pathway is absolutely required for proper nephrogenesis as ablation of *Wnt4* leads to kidney agenesis^56^ and a low level of WNT signal is necessary for maintaining the progenitor pool^41^. However, excessive WNT stimulation causes early cessation of nephrogenesis and NPC pool depletion^40,41^. Interestingly, WNT signaling depends on lipid raft integrity to function, and Wnt molecules require specific lipidation to be functional^20,50,57,58^. Acetyl-CoA is necessary for both cholesterol synthesis which in turn is involved in the formation of lipid rafts^59^, that facilitate WNT signal transduction^58,60^ and fatty acid synthesis which is necessary for lipidation of WNT molecules^61^. Thus, it is possible that glycolysis inhibition may have favored differentiation by disrupting the low levels of WNT signaling required for NPC “stemness” maintenance^41^. In support of this view, treatment with statins has been shown to reduce WNT signaling in colon cancer cells^50^.

Furthermore, acetyl-CoA formation is directly proportional to the cellular glycolytic flux, as glucose is a precursor of acetyl-CoA^29^. Glycolytic flux is controlled at several steps inside cells. We and others have shown that the inhibition of PI3K pathway decreases glycolytic flux and potentiates differentiation in nephron progenitor cells^13,62–65^. Treatment with YN1 decreased the expression of several genes, including Pi3kca, Akt1 glycolytic enzymes such as the pyruvate kinase isoform M2 (PKM2) (Supplementary Fig.1) which is encoded by the *Pkm* gene, and is expressed exclusively in embryonic stages, proliferating cells, and cancer cells^66^. PKM2 has been shown to transactivate beta-catenin in response to PI3K/ERK activation^67,68^ and a recent study has found that MAPK/ERK pathway (target of PI3K) regulates fate decisions in embryonic kidney mesenchyme cell line^30^. Thus, alterations in glucose metabolism can disturb the complex network of metabolites and signaling molecules that govern kidney development.

Perhaps the most important finding of our study is that sodium acetate supplementation can nourish kidney development and rescue glycolysis inhibition by YN1. Acetate can be easily converted to acetyl-CoA inside cells (and vice-versa). Acetate can be generated through various processes such as reactions of deacetylation, Acetyl-CoA transferases, or alcohol consumption, and as a by-product of dietary fiber fermentation by the gut microbiome^55,69–71^. It is likely that the latter process accounts for the vast amount of circulating acetate. Investigating the extent to which these alternative sources of acetate impact kidney development may pave the way for new preventive actions toward offset low nephron endowment at birth, a major cause of hypertension and chronic kidney disease later in life^4,72^. In NPC, acetate metabolism could be facilitated by Acetyl-CoA synthetase enzymes (ACSS1, ACSS2 and ACSS3). Indeed, we detected low-level expression of Acss2 mRNA present at E13.5 (Supplementary Fig.1 and Supplementary Data 1 and 2) but we failed to detect Acss2 with specific antibodies in NPCs at later stages. Also, acetyl-CoA production could be generated by the occurrence of TCA enzymes, such as the pyruvate dehydrogenase complex (PDH) in the nucleus, as shown previously^73,74^. Whichever the case, the current evidence supports the hypothesis that high levels of acetyl-CoA in the nucleus and cytosol favor growth or cellular-responsive states which is consistent with pleiotropic effects of acetyl-CoA^26,42,73,75–77^.

Acetate/acetyl-CoA could favor NPC maintenance via epigenetic regulation as shown for embryonic stem cells in which glycolysis-generated acetyl-CoA drives histone acetylation during pluripotency, while reduction of glycolysis results in decreased acetyl-CoA availability, deacetylation, and differentiation^26^. In line with these thoughts, a recent report showed that a high-fiber diet or sodium acetate supplementation can inhibit histone deacetylase activity in-vivo and ameliorate the effects of acute kidney injury^78^ indicating, that acetate may play an epigenetic role in kidney cells that goes beyond development. In the future, it will be interesting to investigate whether and how acetate/acetyl-CoA impacts histone acetylation and chromatin accessibility in NPC and whether acetate supplementation can increase NPC poll in vivo.

Finally, previous studies have shown that congenital anomalies of the kidney and urinary tract (CAKUT) account roughly for 20 to 30 percent of all anomalies identified in the prenatal period. CAKUT plays a causative role in 30 to 50 percent of cases of chronic kidney disease requiring kidney replacement therapy in children^79,80^. To date, only 5–20% of all CAKUT cases can be explained by monogenic abnormalities^81,82^. Therefore, many CAKUT cases remain unexplained. Current evidence suggests that epigenetic alterations may account for kidney development abnormalities in CAKUT^83^. Cellular metabolism is emerging as a missing link between normal and abnormal development^16,17,23,27,83^. To date, only a handful of studies have investigated how cell metabolism interferes with kidney development. For instance, a recent study showed that either methionine supplementation or activation of the mTOR pathway rescues the reduction in nephron endowment in the offspring of mice fed a hypocaloric diet^14^. Iron supplementation during gestation was shown to increase nephron number in dams fed an iron-poor diet^84–86^. Here, we present the consequences of abnormal glucose metabolism during kidney development. Our study has a larger spectrum of applications, such as formulation of prenatal diets and supplements, diet formula composition for preterm babies (often born with low nephron endowment), in-vitro growth of kidney organoids, kidney-specific cell-type growth and differentiation, and regenerative developmental nephrology. Altogether, our data identify Acetate as a key metabolite for normal kidney development. Reductions in acetyl-CoA metabolism hinder normal kidney development, whereas supplementation stimulates it.

## Methods

### IACUC statement

Animal protocols utilized in this study were approved by and in strict adherence to guidelines established by the Tulane University Institutional Animal Care and Use Committee.

### Animals

CD1 outbred mice were acquired from Charles River Laboratories and bred in-house at our SPF facility. The mice were kept in a standard microisolator cage. We use a 12-hour lights on/12-hour lights off cycle. The temperature was kept between 70–72 °F ( ~ 21 °C) with 40–60% humidity. All females utilized for this study were between 5 and 8 weeks old, and only litters of a first pregnancy were collected. Transgenic or wild-type male mice utilized here ranged from 6 weeks old to 6 months old. For our studies, sex was not considered a biological variable. There is no evidence in the literature to support that sex is an important biological variable in E12.5 E13.5 and P0 mouse offspring regarding the impact of metabolism on kidney development, and thus information regarding sex as a biological variable has not been collected. *Acly* floxed animals were acquired from the Jackson Laboratory, stock #030772. We followed the genotyping strategy indicated with this stock line. Primer For - CCC TCA GAA GGT CAG AGA ACA; Primer Rev- CAG CAG GAG AGC TAG GAC CA. PCR product size => WT = 158 bp. Floxed 334 bp (loxP sites flank exon 9). The strategy to originate this line has been previously published^87^. Six2^GFP-Cre^ transgene mice were acquired from the Jackson Laboratory, stock #009606. Cre PCR genotyping was carried out as previously described^88^. Primer For- TCC-AAT-TTA-CTG-ACC-GTA-CAC-CAA; Primer Rev – CCT-GAT-CCT-GGC-AAT-TTC-GGC-TA. PCR product size = 540 bp (primers bind to core CRE sequence). The transgene function and expression pattern have been described elsewhere^45^. The breeding strategy utilized to generate embryonic kidneys with *Acly* gene deletion in Six2-expressing cells is described in the results section.

### NPC isolation, expansion, and pharmacological treatments

Five female CD1 mice were time-paired with Six2GFP^+^ or GFP^-^ males for E13.5 embryonic kidney harvest (on average 10 embryos per female were collected). E13.5 NPC were isolated by Magnetic Assisted Cell Sorting (MACS) and expanded in Nephron Progenitor Expansion Medium (NPEM), as previously described^64^. After 2–3 passages, NPCs were cultured in differentiation media either with CHIR99021 (Sigma or YN1 (Sigma, cat# SML0947) or UK5099 (Sigma, Cat# PZ0160). The differentiation media was made with DMEM + 1% KO serum replacement (ThermoFisher, cat# 10828028), 200 ng/mL Fgf2 (R7D systems, cat# 3718-GMP), 3.5 µM CHIR99021 (Tocris, cat #4423) or in differentiation media replacing CHIR with 20 µM YN1 (Sigma SML0947) or UK5099 (Sigma PZ0160). After 24 h treatment NPC were harvested for quantitative proteome profiling by TMT LC-MS or transcriptome profiling by bulk RNASeq or Seahorse respirometry (described below). Each plate contained on average 1 × 10^6^ cells. Gene ontology and gene enrichment pathway analysis were performed according to the GSEA workflow (www.gsea-msigdb.org/gsea/index.jsp) with data obtained from 3 biological replicates.

### Extracellular flux measurements (Seahorse respirometry)

E13.5 kidneys were dissected, and the NPC fraction was isolated and cultured as described above. For extracellular flux measurements, cells were seeded 24 h before the assay, at 100,000 cells per well, on Matrigel-coated Seahorse microplates for flux measurements on the XFe24 Extracellular Flux Analyzer (Agilent Seahorse Technologies). Cells were exposed to overnight pharmacological treatment as indicated in the legend of the figures. The media was changed before the assay with the indicated manufacturer’s media. Optimal cell density was determined empirically, based on attaining flux measurements in the linear range and previously published by our group^13^. The glycolysis (Seahorse XF Glycolytic Rate Assay Kit cat# 103344), mitochondrial stress (Seahorse XF Cell Mito Stress Test Kit cat# 103015), and ATP production assay tests (Seahorse XF Real-Time ATP Rate Assay Kit (cat# 103592) all from Agilent, were performed to measure glycolysis and OxPhos and ATP production respectively, according to manufacturer recommendations. All measurements were recorded from three to five biological replicates with three technical replicates per experiment. All data were normalized to cell numbers.

### Tandem mass tag mass spectrometry

The steps of protein extraction and quantitative proteome profiling by TMT LC-MS were performed at the Proteomics Core Facility-LSU School of Medicine. Protein extracts were isolated from NPC treated with vehicle or CHIR or YN1. These experiments have been performed with 3 biological replicates. Next, the protein extracts were reduced, alkylated, and digested overnight. Samples were labeled with the TMT Reagents and then mixed before sample fractionation and clean up. Labeled samples were analyzed by high-resolution LC-MS/MS on a Thermofisher Fusion Orbitrap. Data collection was repeated for a total of 3 technical replicates. Data analysis to identify peptides and quantify reporter ion relative abundance was completed using Proteome Discoverer version 2.2.0.388 (Thermofisher Scientific).

The MS/MS raw files were processed by the SEQUEST algorithm in which a search was performed against protein FASTA Database Mus musculus (SwissProt TaxID=10090) Version: 2017-10-25 concatenated with a reverse protein sequence decoy database. Static modifications included TMT reagents on lysine and N-termini (+229.163 Da) and carbamidomethyl on cysteines (+57.021 Da). Dynamic Modifications included Oxidation of Methionines (+15.995 Da), Phosphorylations of Serine, Threonine, and Tyrosine (+79.966 Da), and N-Terminal Acetylation (+42.011 Da). Precursor mass tolerance was 10 ppm fragment mass tolerance was 0.6 Da, and the maximum number of missed cleavages was set to 2. Only high-scoring peptides were considered utilizing a false discovery rate of <1%, and only one unique high-scoring peptide was required for the inclusion of a given identified protein in our results.

The target-decoy strategy was used to evaluate the false discovery rate (FDR) of peptide and protein identification. To remove false positive matches and low-quality peptide spectrum matches (PSM) a filter by minimal peptide length (7 amino acid), m/z accuracy (e.g., 5 ppm), and matching scores (J score and deltaCn) was applied. Further filtered by matching scores to reduce protein FDR to below 1%. The proteins were quantified by summing reporter ion counts across all matched PSMs. The average of all reporter ions was used to calculate a relative signal between each reporter ion and the average. The relative signals of PSMs were summed for identified proteins. Finally, these relative signals were converted to absolute signals by multiplying the averaged reporter ion intensity of the top 3 PSMs in corresponding proteins. The mass spectrometry proteomics data have been deposited to the ProteomeXchange Consortium via the PRIDE partner repository with the dataset identifier PXD045445 and 10.6019/PXD045445. The protein quantification values were exported in Excel for further analysis described below.

### Bulk RNA sequencing

The Bulk RNA sequencing experiments were performed at Tulane Center for Translational Research in Infection & Inflammation: NextGen Sequencing Core. Total RNA was isolated using Qiagen RNeasy Mini-kit (cat# 74104) with on-column DNase digestion. Samples were sent to Genewiz and passed quality control. RNA sequencing was non-strand specific and sequenced on Illumina-HiSeq 2×150 bp per lane. The RNA library was prepared with poly-A tail selection. Before starting alignment and differential expression FASTQ files are checked for quality control using FASTQC. Alignment of the reads was done using STAR^89^ to the mm9 and mm10 genomes. This produced wiggle files for visualizing tracts. RSEM package was used to quantify read counts and normalized counts to create a relative molar concentration for the reads from the FASTQ files^90^. The transcript abundance estimation (estimated counts and TPMs-transcripts per million) was obtained by aligning reads to the reference transcriptome using Kallisto. For each sample, read counts were collapsed from transcripts to genes and then a count matrix was prepared. These experiments have been performed with three biological replicates.

### Organ culture

E12.5 kidneys were harvested from CD1 mice and cultured in complete media as described previously with slight modifications^13^. Briefly, culture media contain DMEM/F12 (ThermoFisher, cat# 12634028), 3% FBS (GIBCO, cat# A3840002), and 1x penicillin/streptomycin at 37 °C, 5% CO_2_ and pH 7.4. Contralateral kidneys were treated with either vehicle or 10 µM Pravastatin (Tocris, cat#2318) or 20 µM YN1 without or with 10 mM Sodium acetate (Sigma-Aldrich, cat# 71196) or 10 µM UK5099 for 24–48 h in 0.4 µm transwell plates (Greiner, cat#657640). Stock solutions of Sodium acetate (100X in PBS pH 7.4), Pravastatin, YN1, UK5099 (1000X in DMSO), or vehicle were diluted directly in the culture media, then the solution was left in the tissue culture incubator (5% CO_2_), at 37 °C for 10 min and the pH was measured before each experiment. No pH variations were detected after adding any specified compound.

### Whole-mount immunofluorescence staining

Kidneys were fixed in 4% paraformaldehyde and processed for immunofluorescence staining in Tris-Saponin buffer, described before^13^. Antibodies against the following proteins were used:

1. Mouse IgG Anti-Lhx1 (Developmental Hybridoma, 4F2) (1:100 v/v) + TSA Detection (Donkey anti-Mouse IgG1 Secondary Antibody, HRP, Jackson Immuno Research™ (cat # 715-036-150) (1:100 v/v)
2. Rabbit IgG Anti-Acly (Proteintech, cat # 15421-1-AP) (1:200 v/v) + Donkey anti-Rabbit IgG Highly Cross-Adsorbed Secondary Antibody, Alexa Fluor™ 488 (cat # A-21202) (1:400 v/v)
3. Rabbit IgG Anti-Six2 (Proteintech, cat# 11562) (1:200 v/v) + Donkey anti-Rabbit IgG Highly Cross-Adsorbed Secondary Antibody, Alexa Fluor™ 555 (cat # A-31572) (1:400 v/v)
4. Goat IgG Anti-Wnt4 (R&D systems, cat # AF475) (1:100 v/v) + TSA Detection (Donkey anti-Goat IgG1 Secondary Antibody, HRP, Jackson Immuno Research™ (cat # 705-036-147) (1:100 v/v)
5. Mouse IgG Anti-Cytokeratin Pan (Sigma, cat# C2562) (1:200 v/v) + Donkey anti-Mouse IgG Highly Cross-Adsorbed Secondary Antibody, Alexa Fluor™ 647 (cat # A-311571) (1:200 v/v)
6. Chicken IgG Anti-GFP (Abcam, #cat ab300643) (1:200 v/v) + Donkey anti-Mouse IgG Highly Cross-Adsorbed Secondary Antibody, Alexa Fluor™ 555 (cat # A-21437) (1:400 v/v)
7. Fluorescent-tagged Dolichos Biflorus Agglutinin (DBA) (Vector Labs, cat# FL1031) (1:100 v/v) was used for UB-specific lectin staining.
8. Hoechst-33342 (ThermoFisher, cat# 62249) was used for nuclear staining with 1:10.000 v/v dilution.

### Fluorescence-activated cell sorting

E12.5 Six2^GFP-Cre^ kidneys were cultured in the presence or absence of either vehicle, sodium acetate, pravastatin, YN1 or CHIR accordingly. Past 24 h in culture, the NPCs were isolated as described above and sorted using Fluorescence-Activated Cell Sorting (FACS). Standard FACS parameters were directly obtained along sides with sorting, such as cell size, GFP intensity, and percentage of cells positive for GFP per kidney, as described in the results section and in the legend of specific figures.

### Nascent nephron counts

For Lhx1+ nascent nephron counting in organ culture experiments, 19 paired kidneys from four litters were used for 24-hour treatment and control. Paired two-tailed paired t-test was used to calculate p-values, with p < 0.05 considered significant.

### Six2+ cells counting in Acly Cap-mesenchyme-specific deletion

P0 kidneys were paraffin-embedded and sectioned (5 µm). Sections were processed for immunofluorescence as previously described by our group^13^. Six2 positive cells were counted per niche at 40X magnification. The number of sections counted is specified in the results sections per group (control, heterozygous, and homozygous mutant kidneys). The R package ggstatplot^91^, was used for descriptive statistics and statistical tests for differences between groups. Fluorescence intensity was quantified in P0 sections of control and mutant kidneys using the image processing analysis in Java application from NIH, ImageJ^92^.

### Acetyl-CoA measurements

To determine whether sodium acetate treatment was sufficient to increase intracellular concentrations of Acetyl-CoA forty-six E13.5 kidneys were split into two groups (Control or Sodium acetated treated). These two groups were cultured for 24 h in presence of 10 mM sodium acetate or vehicle. Then, the kidneys were harvested and used to measure the acetyl-CoA level with the acetyl-coenzyme A assay kit (Sigma-Aldrich, MAK039) according to the manufacturer’s protocol.

### Statistics & reproducibility

No statistical method was used to predetermine the sample size for the RNA seq and Proteomics experiments. Details of sample preparation are presented for each experiment in the paper as a subsection of Methods. No data were excluded from the analyses. The experiments were not randomized. The Investigators were not blinded to allocation during experiments and outcome assessment. Statistical analysis and plotting were performed in R programming language (v4.1.1) and the results were plotted with the following libraries:

1) ggplot2 (ggplot2.tidyverse.org/); 2) ggpubr (github.com/kassambara/ggpubr/); 3) ggstatsplot (https://indrajeetpatil.github.io/ggstatsplot/).

### RNA sequencing analysis

To perform RNA sequencing analysis, we utilized a count matrix containing expression values measured in transcript per million (TPM). This count matrix served as the input for Sleuth, an online differential gene expression analysis tool (http://pachterlab.github.io/sleuth)^93^. When comparing two groups, Sleuth employs the Wald test as its default hypothesis testing method. To control for multiple comparisons, two correction methods are applied: the Wald test q-value and the Likelihood Ratio Test (LRT) q-value. These correction values are included in the processed files. For further filtering, the differentially expressed genes were defined with the following threshold: log fold change > 0.6, *p*-value < 0.05.

### Proteomics analysis

We conducted proteomics analysis using the abundance table (described above), which included a pairwise comparison of each sample to the Control NPC in NPEM. To assess the differences between the two groups, we employed a two-sided t-test. The associated p-values, as well as absolute and relative abundance quantifications, are publicly available on Figshare (10.6084/m9.figshare.20459790.v1). Proteins were considered to exhibit distinct abundance levels between the control and treatment groups when the p-value was less than 0.05 and the abundance ratio (the ratio of any pairwise comparison = treatment/control) was greater than 1.1 for upregulated proteins and the abundance ratio lesser than 0.9 for downregulated proteins.

### Gene enrichment analysis

We conducted gene enrichment analysis using the online tool EnrichR with default settings (https://maayanlab.cloud/Enrichr/) and BioJupies^94^. For this analysis, we provided specific gene lists derived from our DEG analysis. Specifically, we focused on terms related to developmental processes and metabolism. The results of this enrichment analysis, using the gene set Gene Ontology have been included as Supplementary Data files. For Figs. 2 and 3, we took the DEGs between control *vs* YN1 or CHIR and confirmed the top terms with different gene sets (Gene Ontology Biological Processes, Panther, KEGG, Reactome, MSigDB_Hallmark and Wikipathways). Then we filtered for common terms in all gene sets, and further filtered for terms associated with metabolism and developmental processes. The final resulting tables used to build the bar graphs in Figs. 2 and 3 are provided as sourced data in the Source Data file. For Fig. 3e we applied further filtering criteria. We selected genes with a log fold change (LogFC) less than −0.6 and a p-value less than 0.05, designating these genes as downregulated genes. Subsequently, this refined list of downregulated genes was used as input for the EnrichR application, as described above. The outcome was a visualization of the top 15 Gene Ontology terms, presented as bar charts based on their -log10(p-value) scores.

The metabolic pathways were plotted with the assistance of the R library “pathview”^95^. The logFC of DEG list obtained from comparisons between Ctrl samples and YN1-treated NPC were used as input. We set p-value < 0.05 as the cutoff threshold. Source data is provided as source data files.

### Reporting summary

Further information on research design is available in the Nature Portfolio Reporting Summary linked to this article.

### Supplementary information

Supplementary Information

Description of Additional Supplementary Files

Supplementary Data 1

Supplementary Data 2

Supplementary Data 3

Supplementary Data 4

Reporting Summary

### Source data

Source Data

## Data Availability

The bulk RNA sequencing series is available at NCBI GEO under the accession # GSE210937. The mass spectrometry proteomics data have been deposited to the ProteomeXchange Consortium via the PRIDE partner repository with the dataset identifier PXD045445 and 10.6019/PXD045445. Relevant processed proteomic files have been deposited with Figshare under the following private link https://figshare.com/s/95a3dea586c92e7150de. Source data are provided with this paper.

## Code availability

This paper has not used any proprietary code. All libraries used for data analysis are publicly available and are described in the “Statistics & Reproducibility**”** sub-section.
