# Supplementary material for: Acetyl-CoA is a key molecule for nephron progenitor cell pool maintenance
Source: Nat Commun. 2023 Nov 25;14:7733. doi: 10.1038/s41467-023-43513-7 (PMC10676360; doi:10.1038/s41467-023-43513-7)
Supplement: Supplementary file 1 — Supplementary Information [file 41467_2023_43513_MOESM1_ESM.pdf]

Supplementary Figures

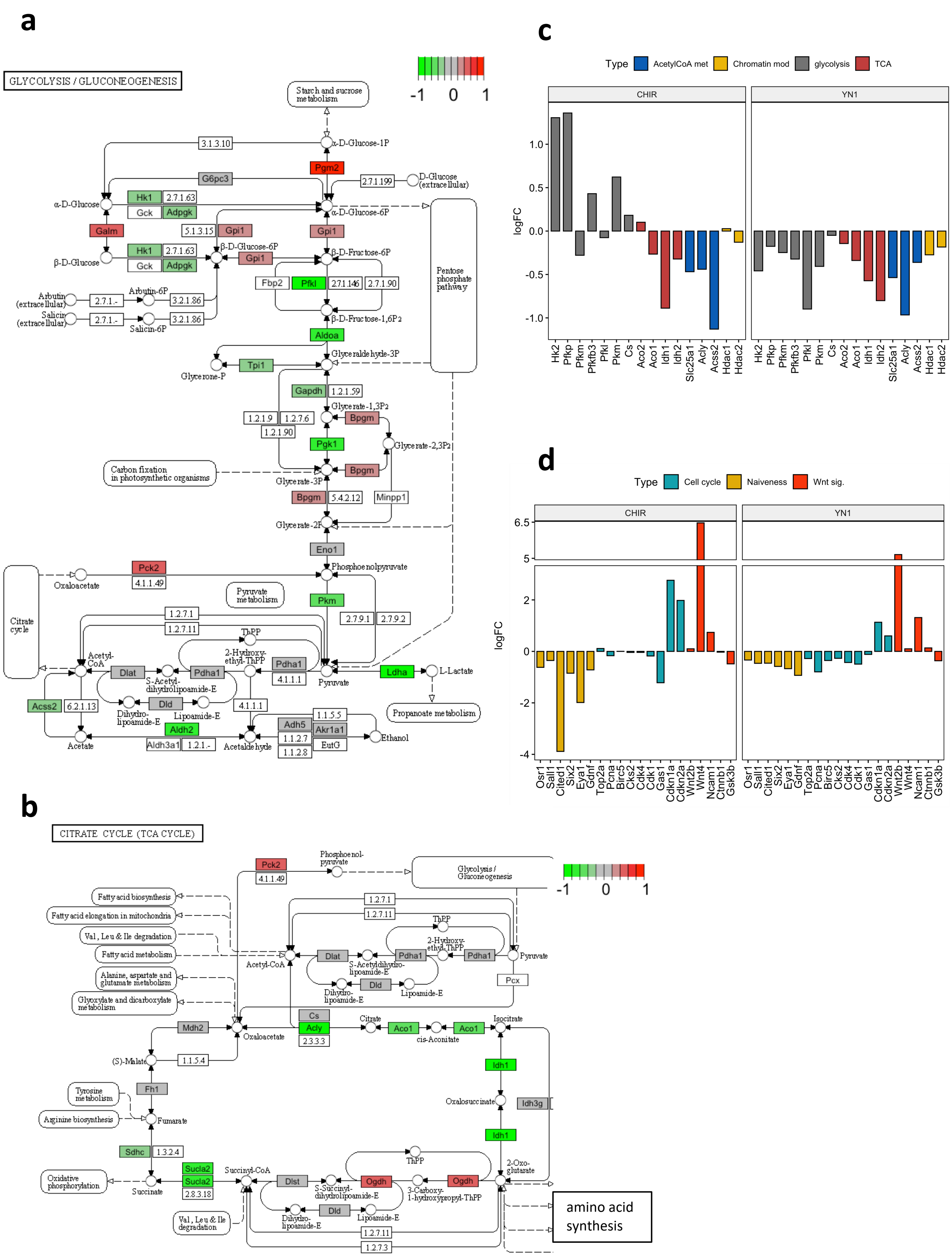
a
GLYCOLYSIS / GLUCONEOGENESIS
-1 0 1
Starch and sucrose metabolism
α-D-Glucose-1P
Pgm2
D-Glucose (extracellular)
G6pc3
α-D-Glucose
Hk1
Gck
Adpgk
α-D-Glucose-6P
Galm
Gpi1
β-D-Glucose-6P
β-D-Glucose
β-D-Fructose-6P
Fbp2
Pfkl
Pentose phosphate pathway
Arbutin (extracellular)
Arbutin-6P
Salicin (extracellular)
Salicin-6P
β-D-Fructose-1,6P2
Aldoa
Glyceraldehyde-3P
Tpi1
Glycerone-P
Gapdh
Glycerate-1,3P2
Bpgm
Glycerate-2,3P2
Pgk1
Carbon fixation in photosynthetic organisms
Glycerate-3P
Minpp1
Glycerate-2P
Eno1
Pck2
Phosphoenolpyruvate
Oxaloacetate
Citrate cycle
Pyruvate metabolism
Pkm
Acetyl-CoA
Dlat
Pdha1
S-Acetyl-dihydrolipoamide-E
2-Hydroxy-ethyl-ThPP
ThPP
Pyruvate
Ldha
L-Lactate
Acss2
Dihydro-lipoamide-E
Dld
Lipoamide-E
Propanoate metabolism
Aldh2
Adh5
Akr1a1
Acetate
Aldh3a1
Acetaldehyde
EutG
Ethanol
b
CITRATE CYCLE (TCA CYCLE)
Pck2
Phosphoenol-pyruvate
Glycolysis / Gluconeogenesis
-1 0 1
Fatty acid biosynthesis
Fatty acid elongation in mitochondria
Val, Leu & Ile degradation
Fatty acid metabolism
Alanine, aspartate and glutamate metabolism
Glyoxylate and dicarboxylate metabolism
Acetyl-CoA
Dlat
Pdha1
2-Hydroxy-ethyl-ThPP
Pyruvate
S-Acetyldihydro-lipoamide-E
Dihydro-lipoamide-E
Dld
Lipoamide-E
Pcx
Mdh2
Oxaloacetate
Cs
Acly
Citrate
Aco1
cis-Aconitate
Isocitrate
(S)-Malate
Idh1
Fh1
Tyrosine metabolism
Oxalosuccinate
Idh3g
Arginine biosynthesis
Fumarate
Sdhc
Sucla2
Succinyl-CoA
Dlst
S-Succinyl-dihydrolipoamide-E
Ogdh
3-Carboxy-1-hydroxypropyl-ThPP
2-Oxo-glutarate
Oxidative phosphorylation
Succinate
amino acid synthesis
Val, Leu & Ile degradation
Dihydro-lipoamide-E
Lipoamide-E
c
Type AcetylCoA met Chromatin mod glycolysis TCA
CHIR
YN1
logFC
Hk2 Pfkp Pfkm Pfkfb3 Pfkl Pkm Cs Aco2 Aco1 Idh1 Idh2 Slc25a1 Acly Acss2 Hdac1 Hdac2
d
Type Cell cycle Naiveness Wnt sig.
CHIR
YN1
logFC
Osr1 Sall1 Cited1 Six2 Eya1 Gdnf Top2a Pcna Birc5 Cks2 Cdk4 Cdk1 Gas1 Cdkn1a Cdkn2a Wnt2b Wnt4 Ncam1 Ctnnb1 Gsk3b

Fig.S1: Molecular characterization of transcriptomes from YN1 and CHIR-treated NPC. To visualize changes in gene expression, the LogFC values between YN1-treated NPCs and control NPCs were scaled between -1 (green, downregulated), 0 (grey, no change), and +1 (red, upregulated), and the scores were plotted on the KEGG Glycolysis/Gluconeogenesis (a), KEGG citrate cycle (b). LogFC values, representing the gene expression changes between YN1-treated and control NPCs for genes associated with acetyl-CoA production and metabolism, were plotted for multiple genes. These values were color-coded according to their pathway signature (c). LogFC values, representing the gene expression changes between YN1-treated and control NPCs for genes associated with cell cycle (gold), NPC naiveness (cyan), or WNT signaling (red) were plotted for multiple genes (d). Source data are provided as a Source Data file.

a

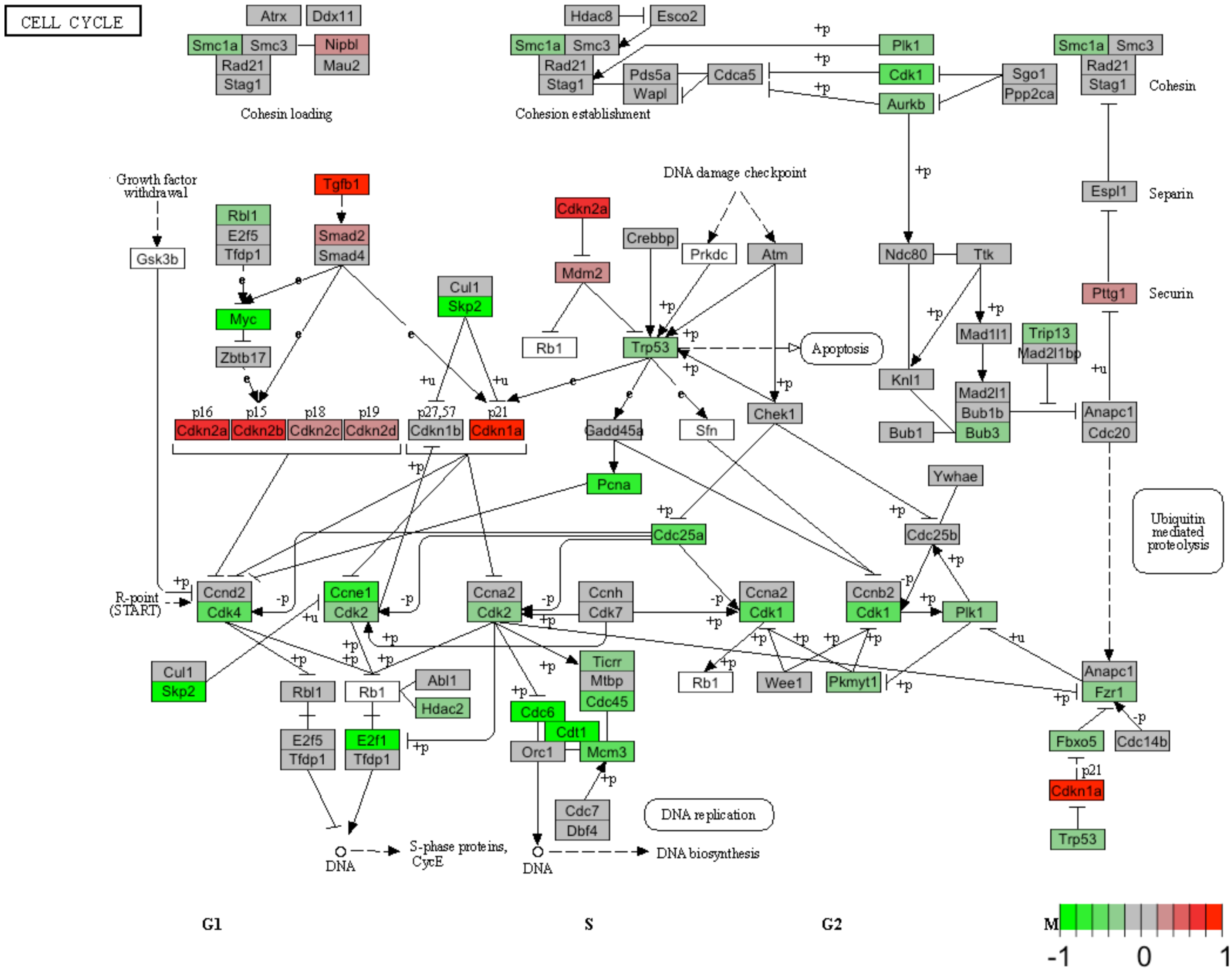
CELL CYCLE
Atrx
Ddx11
Smc1a
Smc3
Nipbl
Rad21
Mau2
Stag1
Cohesin loading
Hdac8
Esco2
Pds5a
Wapl
Cdca5
Plk1
Cdk1
Aurkb
Sgo1
Ppp2ca
Cohesion establishment
Cohesin
Espl1
Separin
Pttg1
Securin
Growth factor withdrawal
Gsk3b
Tgfb1
Smad2
Smad4
Rbl1
E2f5
Tfdp1
Myc
Zbtb17
Cul1
Skp2
Cdkn2a
Mdm2
Rb1
DNA damage checkpoint
Crebbp
Prkdc
Atm
Trp53
Apoptosis
Ndc80
Ttk
Mad1l1
Trip13
Mad2l1bp
Knl1
Mad2l1
Bub1b
Bub3
Bub1
Anapc1
Cdc20
p16
p15
p18
p19
p27,57
p21
Cdkn2b
Cdkn2c
Cdkn2d
Cdkn1b
Cdkn1a
Gadd45a
Sfn
Chek1
Pcna
Ywhae
Ubiquitin mediated proteolysis
Cdc25a
Cdc25b
R-point (START)
Ccnd2
Cdk4
Ccne1
Cdk2
Ccna2
Ccnh
Cdk7
Ccnb2
Abl1
Hdac2
Ticrr
Mtbp
Cdc45
Wee1
Pkmyt1
Fzr1
Cdc6
Cdt1
Orc1
Mcm3
Fbxo5
Cdc14b
Cdc7
Dbf4
DNA replication
DNA
S-phase proteins, CycE
DNA biosynthesis
G1
S
G2
M
-1
0
1

b

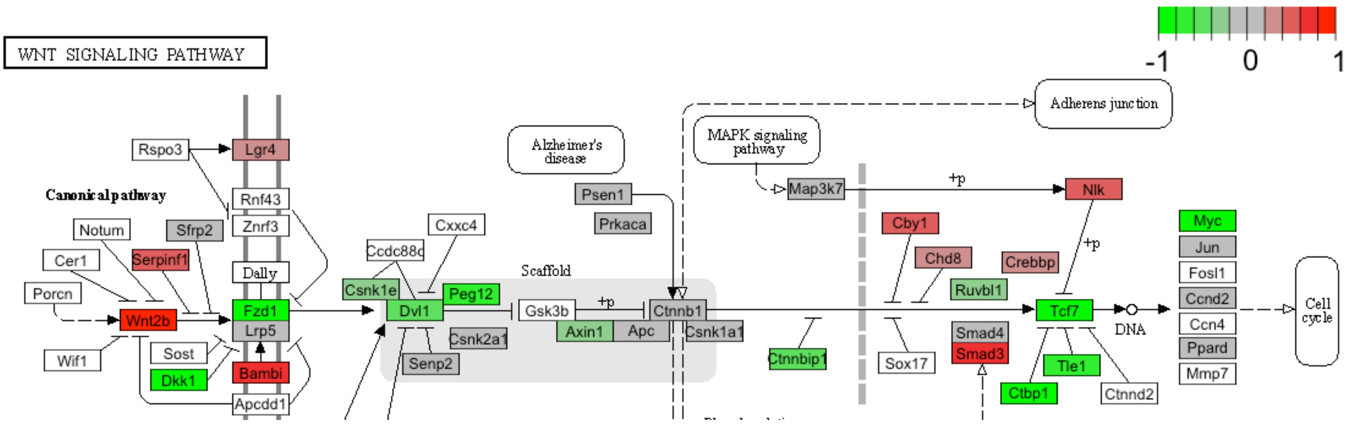
WNT SIGNALING PATHWAY
-1
0
1
Adherens junction
Rspo3
Lgr4
Alzheimer's disease
MAPK signaling pathway
Canonical pathway
Rnf43
Znrf3
Notum
Sfrp2
Cxxc4
Psen1
Prkaca
Map3k7
Nlk
Cer1
Serpinf1
Ccdc88c
Dally
Scaffold
Cby1
Chd8
Crebbp
Myc
Jun
Fosl1
Porcn
Csnk1e
Peg12
Ruvbl1
Ccnd2
Wnt2b
Fzd1
Lrp5
Dvl1
Gsk3b
Ctnnb1
Tcf7
DNA
Cell cycle
Axin1
Apc
Csnk1a1
Smad4
Ccn4
Wif1
Sost
Csnk2a1
Smad3
Ppard
Dkk1
Bambi
Senp2
Ctnnbip1
Sox17
Tle1
Mmp7
Apcdd1
Ctbp1
Ctnnd2

Fig.S2: Impact of YN1 treatment on cell cycle genes and WNT signaling. LogFC values depicting gene expression changes between YN1-treated NPCs and control NPCs were normalized to a color scale ranging from -1 (green, downregulated) to +1 (red, upregulated). These values were then visualized on the KEGG cell cycle pathway (a) and KEGG WNT signaling pathway (b). Notably, the figure highlights the activation of cell cycle inhibitors, including p21 (highlighted in red oval), and a reduction in several genes of the intracellular WNT machinery following YN1 treatment. Source data are provided as a Source Data file.

a

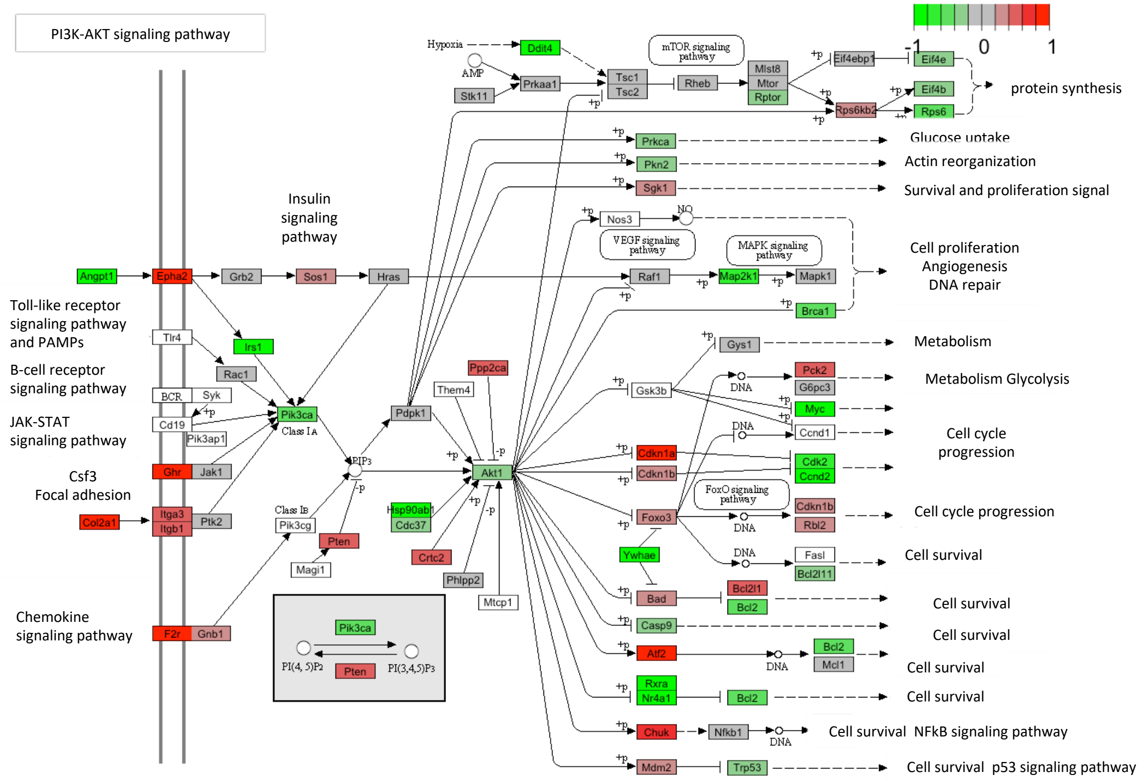
PI3K-AKT signaling pathway
-1
0
1
protein synthesis
Glucose uptake
Actin reorganization
Survival and proliferation signal
Insulin signaling pathway
Cell proliferation
Angiogenesis
DNA repair
Toll-like receptor signaling pathway and PAMPs
B-cell receptor signaling pathway
JAK-STAT signaling pathway
Csf3
Focal adhesion
Chemokine signaling pathway
Metabolism
Metabolism Glycolysis
Cell cycle progression
Cell cycle progression
Cell survival
Cell survival
Cell survival
Cell survival
Cell survival
Cell survival NFkB signaling pathway
Cell survival p53 signaling pathway
Hypoxia
AMP
Ddit4
Prkaa1
Stk11
mTOR signaling pathway
Tsc1
Tsc2
Rheb
Mlst8
Mtor
Rptor
Eif4ebp1
Eif4e
Eif4b
Rps6kb2
Rps6
Prkca
Pkn2
Sgk1
Nos3
NO
VEGF signaling pathway
MAPK signaling pathway
Angpt1
Epha2
Grb2
Sos1
Hras
Raf1
Map2k1
Mapk1
Brca1
Tlr4
Irs1
Rac1
BCR
Syk
Cd19
Pik3ap1
Pik3ca
Class IA
Ghr
Jak1
Itga3
Itgb1
Ptk2
Col2a1
F2r
Gnb1
Class IB
Pik3cg
Pten
Magi1
PIP3
Pdpk1
Them4
Ppp2ca
Akt1
Hsp90ab1
Cdc37
Crtc2
Phlpp2
Mtcp1
PI(4, 5)P2
PI(3,4,5)P3
Gys1
Gsk3b
DNA
Pck2
G6pc3
Myc
Ccnd1
Cdkn1a
Cdkn1b
Cdk2
Ccnd2
FoxO signaling pathway
Foxo3
Cdkn1b
Rbl2
Ywhae
Fasl
Bcl2l11
Bad
Bcl2l1
Bcl2
Casp9
Atf2
Bcl2
Mcl1
Rxra
Nr4a1
Chuk
Nfkb1
Mdm2
Trp53

b

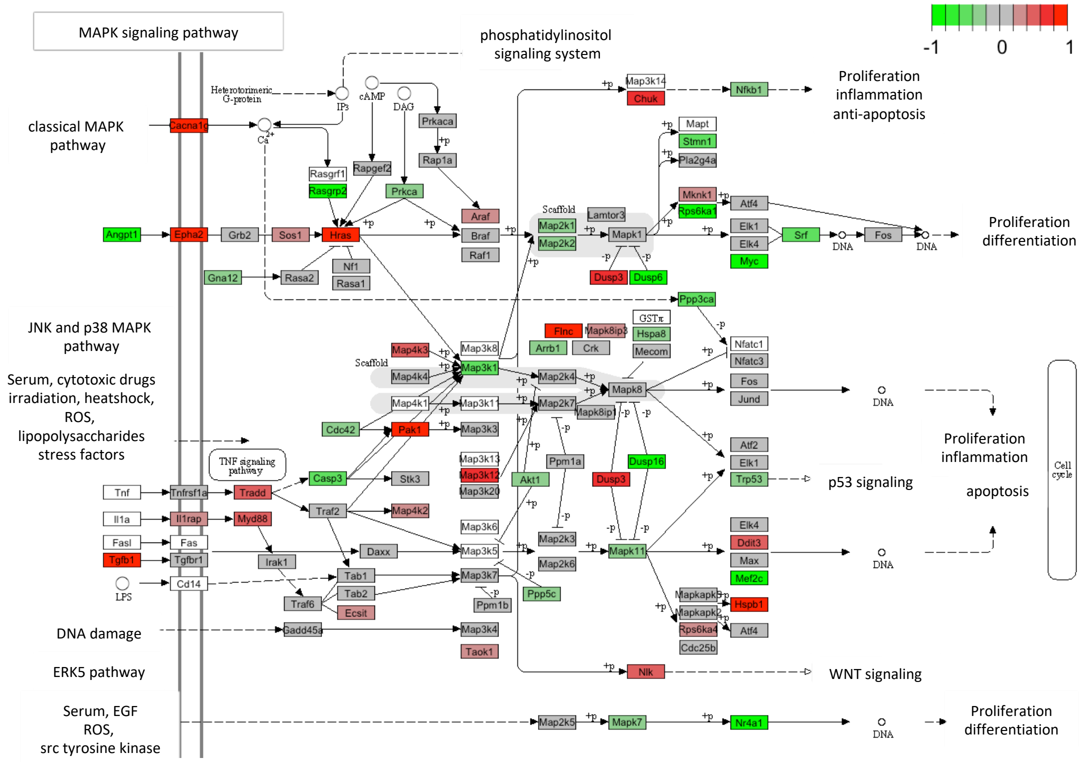
MAPK signaling pathway
phosphatidylinositol signaling system
-1
0
1
classical MAPK pathway
Proliferation inflammation anti-apoptosis
Proliferation differentiation
JNK and p38 MAPK pathway
Serum, cytotoxic drugs irradiation, heatshock, ROS, lipopolysaccharides stress factors
Proliferation inflammation
apoptosis
Cell cycle
p53 signaling
DNA damage
ERK5 pathway
WNT signaling
Serum, EGF ROS, src tyrosine kinase
Proliferation differentiation
Heterotorimeric G-protein
IP3
cAMP
DAG
Ca2+
Cacna1g
Prkaca
Rap1a
Rasgrf1
Rapgef2
Rasgrp2
Prkca
Angpt1
Epha2
Grb2
Sos1
Hras
Araf
Braf
Raf1
Nf1
Rasa1
Gna12
Rasa2
Map3k14
Chuk
Nfkb1
Mapt
Stmn1
Pla2g4a
Mknk1
Rps6ka1
Atf4
Elk1
Elk4
Myc
Srf
Fos
Scaffold
Lamtor3
Map2k1
Map2k2
Mapk1
Dusp3
Dusp6
Ppp3ca
GSTπ
Flnc
Mapk8ip3
Hspa8
Arrb1
Crk
Mecom
Nfatc1
Nfatc3
Fos
Jund
Map4k3
Map3k8
Map4k4
Map3k1
Map2k4
Mapk8
Map4k1
Map3k11
Map2k7
Mapk8ip1
Cdc42
Pak1
Map3k3
Map3k13
Map3k12
Map3k20
Akt1
Ppm1a
Dusp16
Dusp3
Atf2
Elk1
Trp53
TNF signaling pathway
Tnf
Tnfrsf1a
Tradd
Casp3
Stk3
Traf2
Map4k2
Il1a
Il1rap
Myd88
Fasl
Fas
Daxx
Tgfb1
Tgfbr1
Irak1
LPS
Cd14
Tab1
Tab2
Traf6
Ecsit
Map3k6
Map3k5
Map3k7
Ppm1b
Map2k3
Map2k6
Mapk11
Ppp5c
Elk4
Ddit3
Max
Mef2c
Mapkapk3
Mapkapk2
Rps6ka4
Cdc25b
Hspb1
Atf4
Gadd45a
Map3k4
Taok1
Nlk
Map2k5
Mapk7
Nr4a1
DNA

Fig.S3: PI3K and MAPK pathways response to YN1 treatment. LogFC values depicting gene expression changes between YN1-treated NPCs and control NPCs were normalized to a color scale ranging from -1 (green, downregulated) to +1 (red, upregulated). These values were then visualized on the KEGG PI3K-AKT signaling pathway (a) and MAPK signaling pathway (b). The figure highlights a reduction in key elements in these pathways such as PI3K catalytic subunit and AKT1. Source data are provided as a Source Data file.

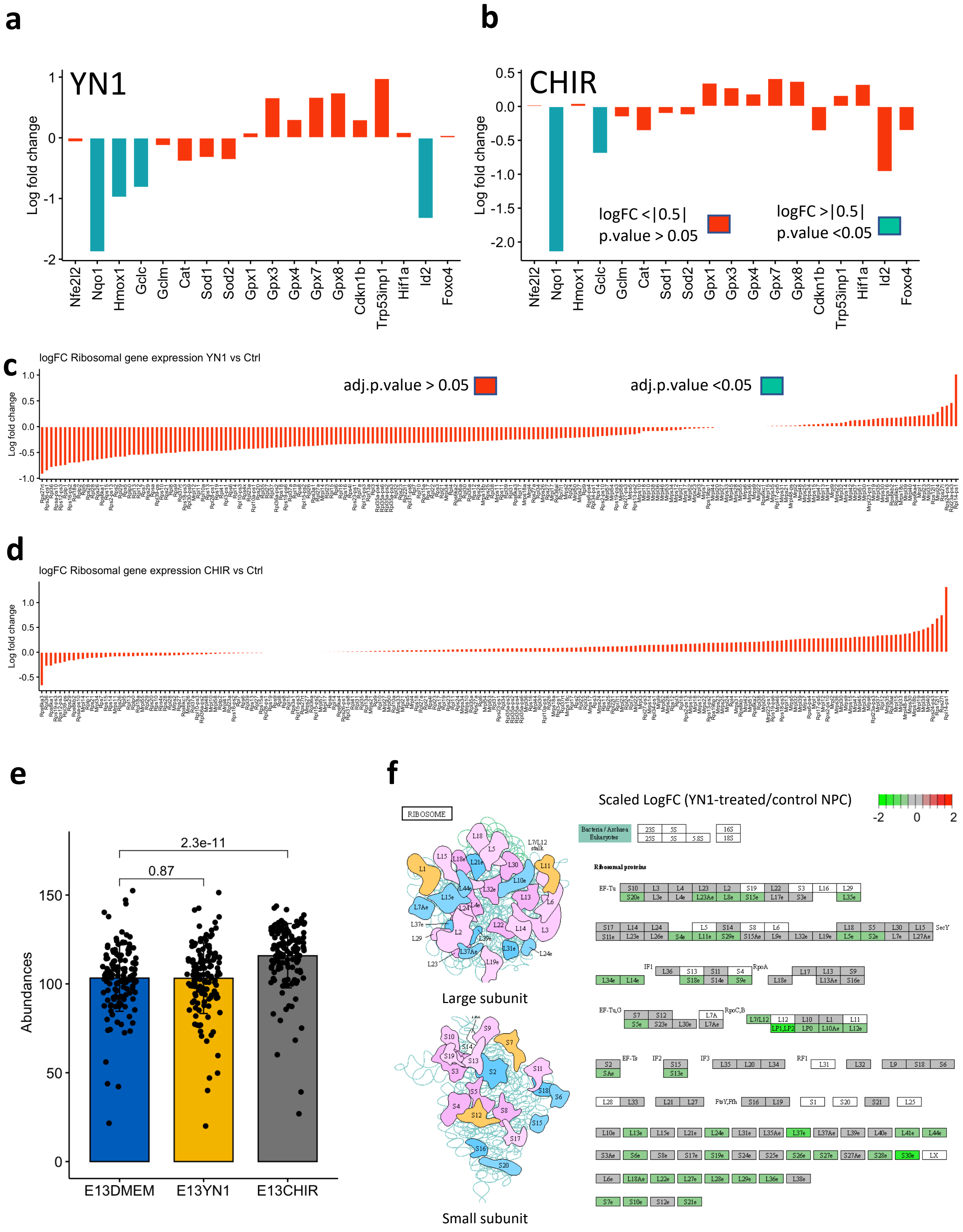
a
YN1
Log fold change
1
0
-1
-2
Nfe2l2
Nqo1
Hmox1
Gclc
Gclm
Cat
Sod1
Sod2
Gpx1
Gpx3
Gpx4
Gpx7
Gpx8
Cdkn1b
Trp53inp1
Hif1a
Id2
Foxo4
b
CHIR
0.5
0.0
-0.5
-1.0
-1.5
-2.0
logFC <|0.5|
p.value > 0.05
logFC >|0.5|
p.value <0.05
c
logFC Ribosomal gene expression YN1 vs Ctrl
adj.p.value > 0.05
adj.p.value <0.05
d
logFC Ribosomal gene expression CHIR vs Ctrl
e
Abundances
2.3e-11
0.87
150
100
50
0
E13DMEM
E13YN1
E13CHIR
f
RIBOSOME
Scaled LogFC (YN1-treated/control NPC)
-2
0
2
Large subunit
Small subunit
Bacteria / Archaea
Eukaryotes
Ribosomal proteins

Fig.S4: Evaluation of stress oxidative response and protein synthesis in the transcriptome of YN1 and CHIR-treated NPC. The LogFC values of genes associated with the oxidative stress response were compared between YN1-treated and control NPCs (a), or CHIR-treated and control NPCs (b) and color-coded based on their LogFC and p-value variation. Only LogFC variations greater than |0.5| and p-values less than 0.05 were considered significant. The LogFC values of ribosomal protein-coding gene expression after YN1 (c) and CHIR (d) treatment are shown. Differences in the abundance of ribosomal proteins after treatment with either YN1 or CHIR compared to control NPC (e). The Kruskal-Wallis test was used for the comparisons across groups and p-values are shown in the figure. To visualize changes in ribosomal protein-coding gene expression, the LogFC values were scaled between YN1-treated NPCs and control NPCs and color-coded as green (downregulated), grey (no change), or red (upregulated), with a scale ranging from -1 to +1. These scores were then plotted on the KEGG Ribosomal pathway (f). Source data are provided as a Source Data file.

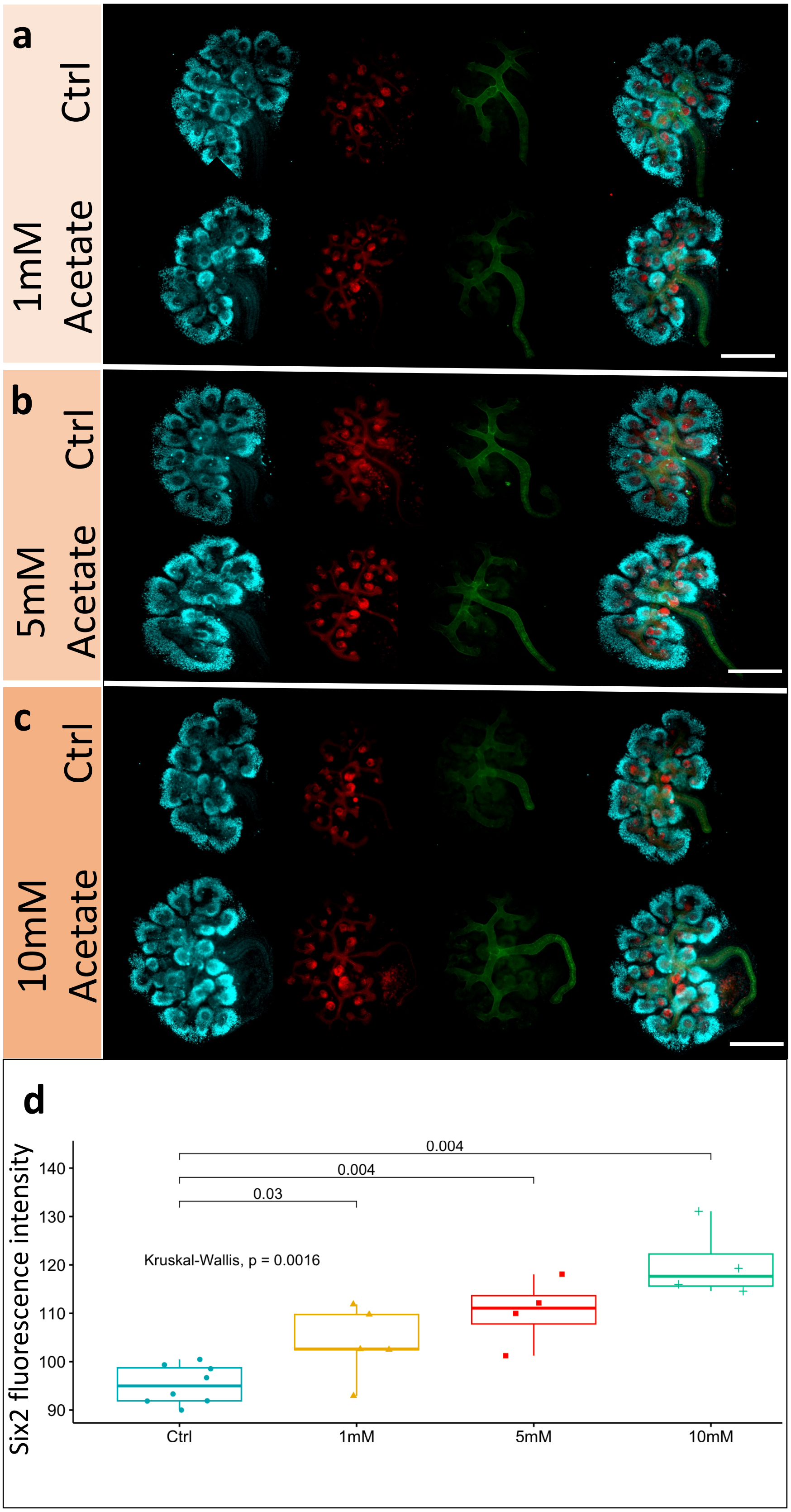
a
Ctrl
1mM Acetate
b
Ctrl
5mM Acetate
c
Ctrl
10mM Acetate
d
Six2 fluorescence intensity
140
130
120
110
100
90
Ctrl
1mM
5mM
10mM
0.004
0.004
0.03
Kruskal-Wallis, p = 0.0016

Fig.S5: Sodium acetate treatment increases CM size. E12.5 mouse kidneys were incubated for 24h in the presence or absence of 1 mM (a), 5 mM (b), or 10 mM sodium acetate (c) and probed with antibodies against Six2 (nephron progenitor cells, Cyan), Lhx1 (nascent nephrons, Red) or DBA (ureteric bud, green). Quantification of Six2 antibody staining, control kidneys n = 8, kidneys treated with sodium acetate 1mM n = 5; 5 mM n = 4; 10 mM n =5) (d). Scale bar 250 µm. Differences between groups were tested with the Kruskal-Wallis test, and Dunn's test for pairwise multiple comparisons, and p-values are reported. Source data are provided as a Source Data file.

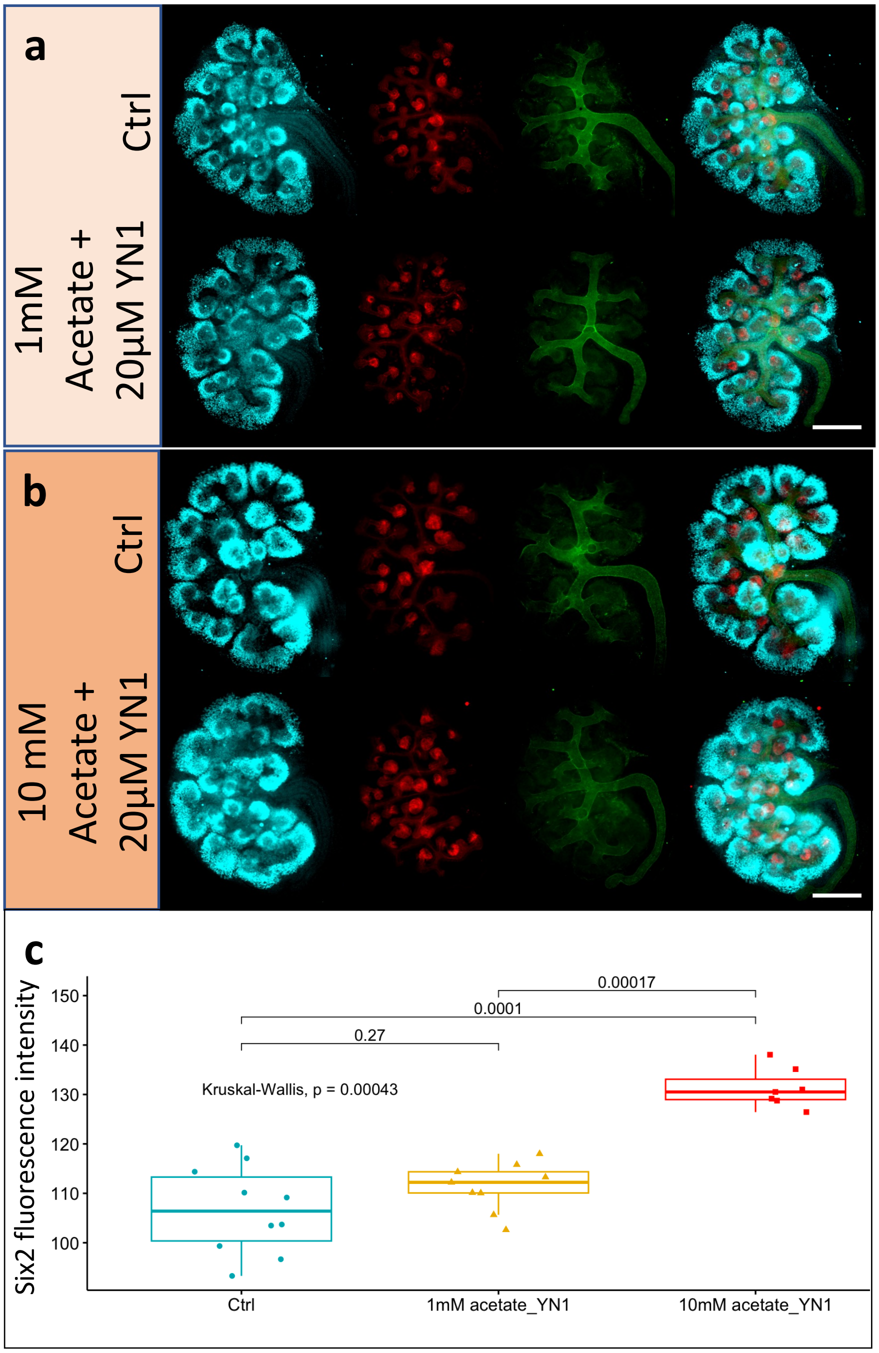
a
Ctrl
1mM
Acetate +
20µM YN1
b
Ctrl
10 mM
Acetate +
20µM YN1
c
Six2 fluorescence intensity
150
140
130
120
110
100
0.00017
0.0001
0.27
Kruskal-Wallis, p = 0.00043
Ctrl
1mM acetate_YN1
10mM acetate_YN1

Fig.S6: Sodium acetate treatment prevents the YN1 effect on developing kidneys. E12.5 mouse kidneys were incubated for 24h in the presence of 20 μM YN1 + either 1 mM (a) or 10 mM sodium acetate (b) and probed with antibodies against Six2 (nephron progenitor cells, Cyan), Lhx1 (nascent nephrons, Red) or DBA (ureteric bud, green). Quantification of Six2 antibody staining, control kidneys n = 10, kidneys treated with sodium acetate 1mM n = 10; 5 mM n = 4; 10 mM n =7) (c). Control kidneys were incubated with vehicle only, scale bar= 250 μm. Differences between groups were tested with the Kruskal-Wallis test, and Dunn's test for pairwise multiple comparisons, and p-values are reported. Source data are provided as a Source Data file.

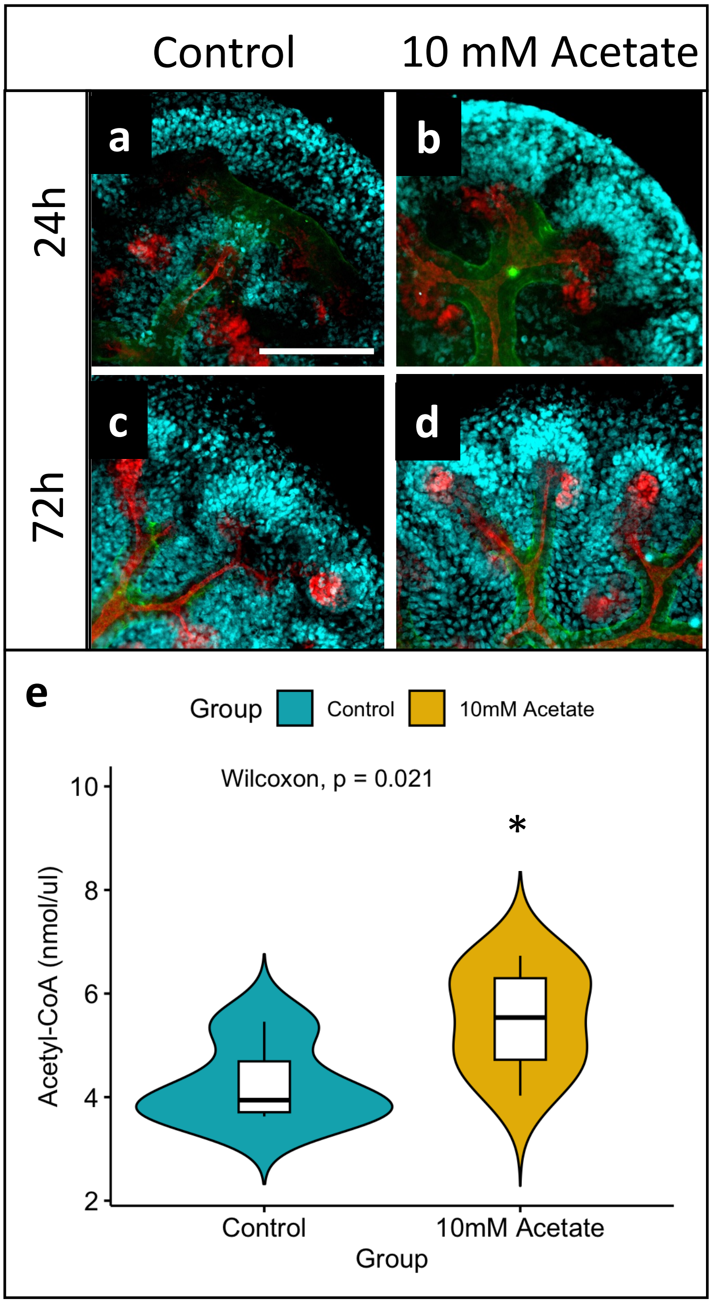

Control
10 mM Acetate
24h
72h
a
b
c
d
e
Group
Control
10mM Acetate
Wilcoxon, p = 0.021
*
Acetyl-CoA (nmol/ul)
10
8
6
4
2
Control
10mM Acetate
Group

Fig.S7: Effects of prolonged exposure to sodium acetate on developing kidneys. E12.5 mouse kidneys were incubated for 24 hours in vehicle (a) or 10 mM sodium acetate (b). E12.5 mouse kidneys were incubated for 72 hours in vehicle (c) or 10 mM sodium acetate (d). Samples were probed with antibodies against Six2 (nephron progenitor cells, cyan), Lhx1 (nascent nephrons, red), or DBA (ureteric bud, green), n = 6 pairs of E12.5 embryonic kidneys per experimental group. The culture media was changed daily until the time of collection at 24 or 72 hours. Scale bar: 100 μm. Sixteen pairs of E12.5 mouse kidneys were incubated for 24 hours in the presence of vehicle or 10 mM sodium acetate, and the cytosolic acetyl-CoA accumulation was measured at the end of the incubation time as described in Methods (e). Differences between groups were tested using the Wilcoxon Rank Sum test with a p-value reported. Source data are provided as a Source Data file.

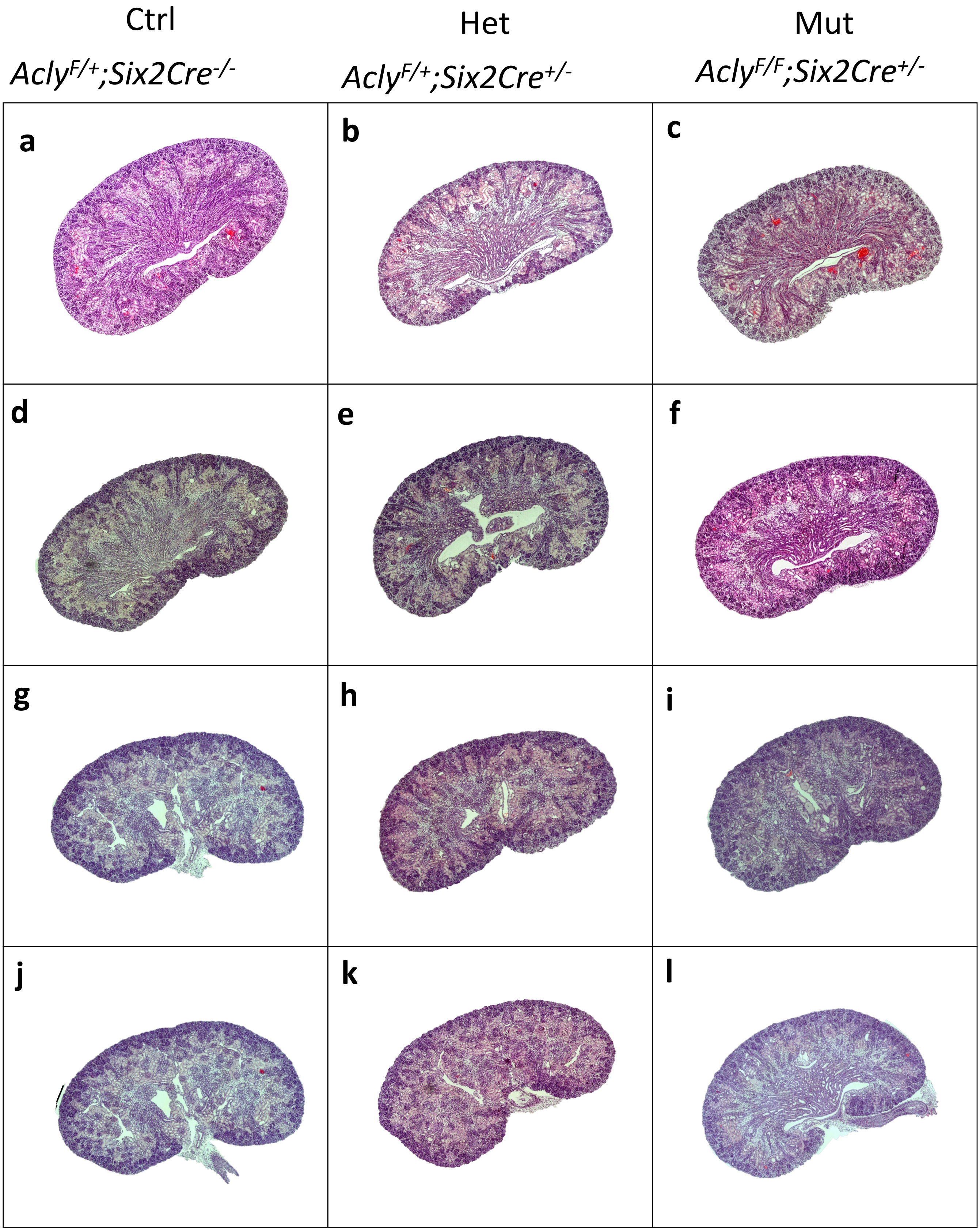
Ctrl
Acly^F/+;Six2Cre^-/-
Het
Acly^F/+;Six2Cre^+/-
Mut
Acly^F/F;Six2Cre^+/-
a
b
c
d
e
f
g
h
i
j
k
l

Fig.S8: Morphological characterization of P0 wild type, heterozygous and mutant kidneys lacking *Acly* in Six2-expressing cells. H&E staining of P0 $Acly^{F/+};Six2Cre^{-/-}$ (control) kidneys (a,d,g,j). H&E staining of P0 $Acly^{F/+};Six2Cre^{+/-}$ (heterozygous) kidneys (b,e,h,k). H&E staining of P0 $Acly^{F/F};Six2Cre^{+/-}$ (mutant) kidneys (c,f,i,l). Notice mutant kidneys with narrower nephrogenic zones and fewer nascent nephrons. N = 4 kidneys per genotype displayed.

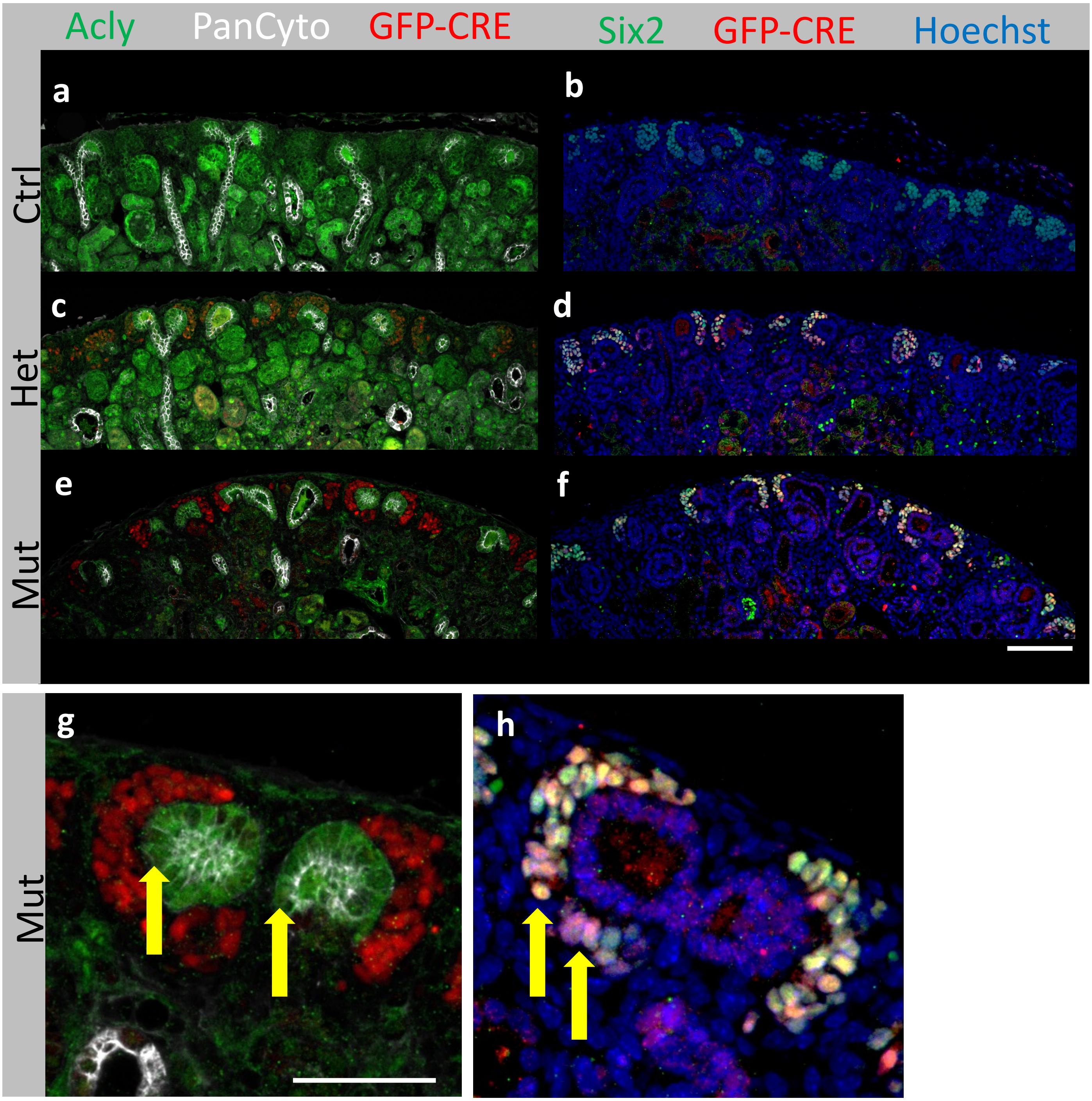

Acly
PanCyto
GFP-CRE
Six2
GFP-CRE
Hoechst
a
b
Ctrl
c
d
Het
e
f
Mut
g
h
Mut

Fig.S9: Removal of Acly from Six2^{+} cells eliminated Acly from the entire nephrogenic lineage. P0 AclyF/+;Six2Cre-/- (control, n=6) kidneys (a,b), P0 AclyF/+;Six2Cre+/- (heterozygous, n=6) kidneys (c,d), and P0 AclyF/F;Six2Cre+/- (mutant n=5) kidneys (e,f) were collected, and analyzed by immunostaining to confirm efficient Acly removal from nephrogenic lineage. Antibodies used and color codes are depicted in the figure. Efficient Acly removal was observed in the mutant kidney (e,f) as evidenced by the absence of green (Acly) signal in the nephrogenic lineage. However, the green (Acly) signal was still present in the UB lineage, and it colocalized with the UB marker Pan cytokeratin (g, yellow arrows). The Cre transgene expression exclusively colocalized with the Six2+ cells (h, yellow arrows), which confirmed the specificity and efficiency of Acly recombination in NPC. A-F scale bar = 250 μm, g,h scale bar = 100 μm. Nuclear staining was carried out with Hoechst 33342.

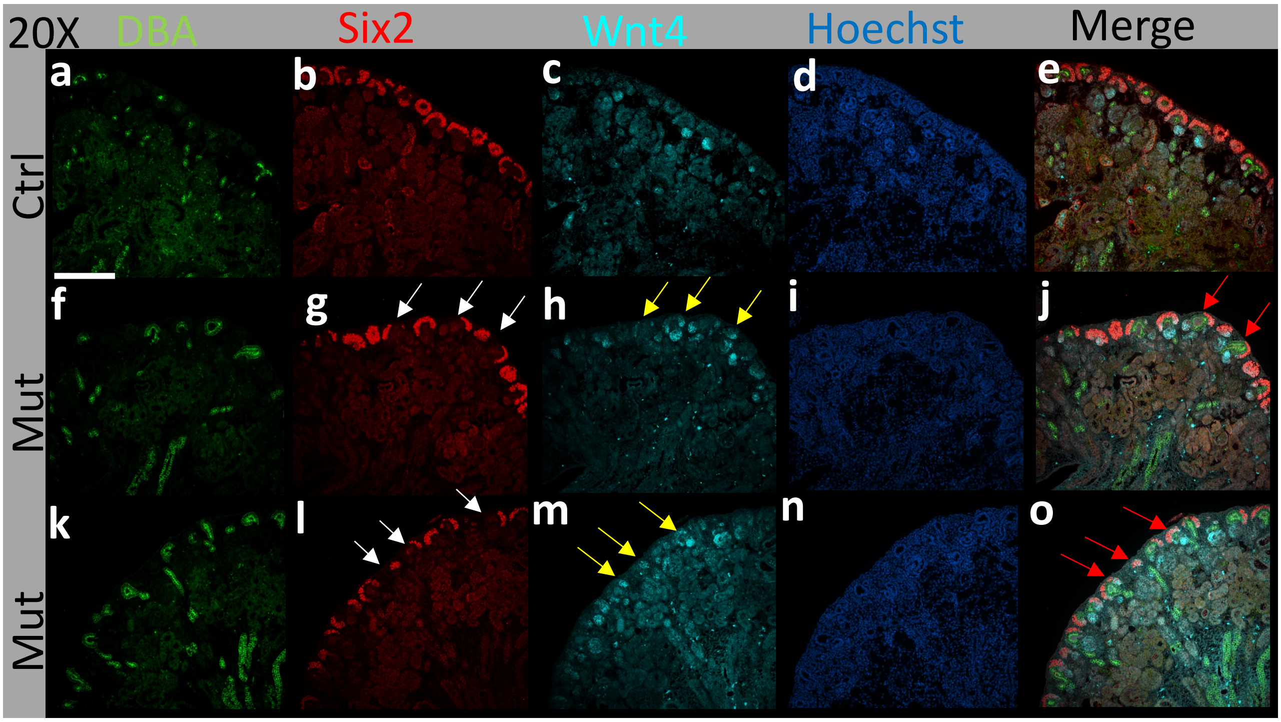
20X
DBA
Six2
Wnt4
Hoechst
Merge
Ctrl
Mut
Mut
a
b
c
d
e
f
g
h
i
j
k
l
m
n
o

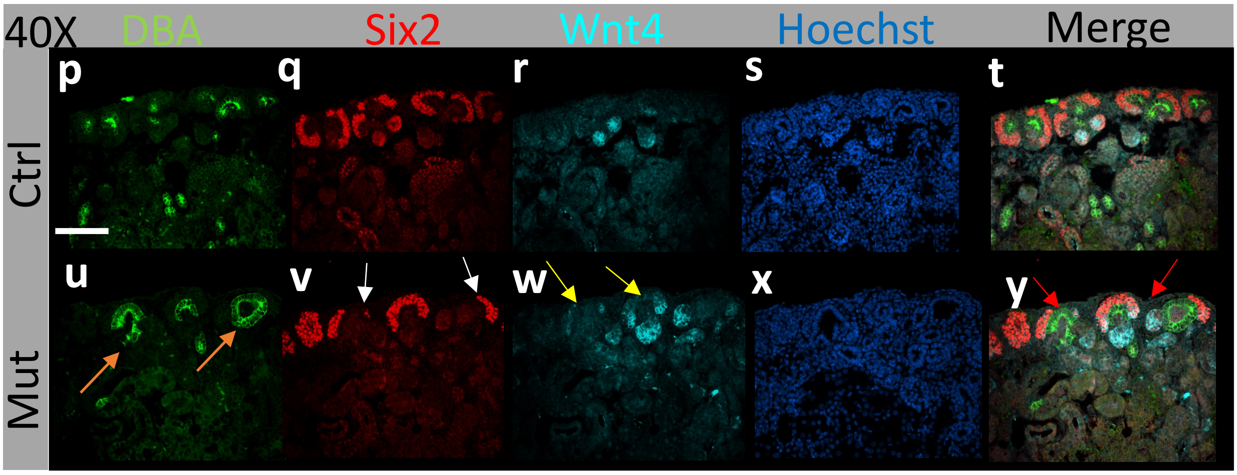
40X
DBA
Six2
Wnt4
Hoechst
Merge
Ctrl
Mut
p
q
r
s
t
u
v
w
x
y

Fig.S10: Immunofluorescence staining of kidney developmental makers in Acly mutant CM at P0. 20X visualization of the nephrogenic zone of control kidneys (Ctrl) (a-e) and two mutant kidneys (f-o) lacking expression of *Acly* in the CM (Mut, mutant kidney). Orange arrows point to dilated UB ampullae; white arrows show areas with depleted cap-mesenchyme; yellow arrows show expansion of Wnt4 towards the cap-mesenchyme. Red arrows show areas co-stained with Wnt4 and Six2 antibodies. On the second panel, a 40x close-up of Ctrl (p-t) and Mut (u-y). These experiments were repeated with at least 5 kidneys per genotype with similar results. UB was stained with DBA (green); CM was stained with Six2 antibody (Red); nascent nephrons and differentiating NPC were stained with Wnt4 (Cyan); Nuclear staining was carried out with Hoechst 33342 (blue). Scale bar a-o = 500 µm; p-y scale bar = 250 µm.

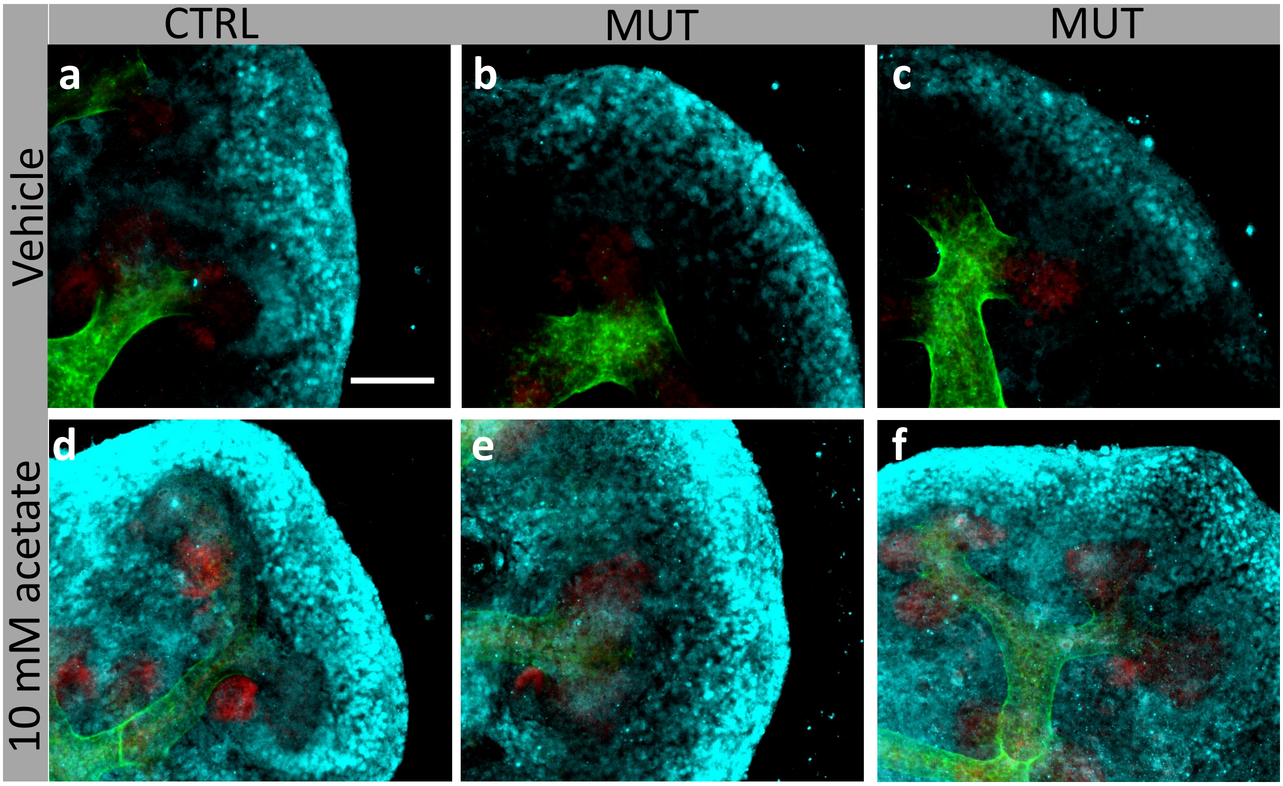
CTRL
MUT
MUT
Vehicle
10 mM acetate
a
b
c
d
e
f

Fig.S11: Acetate supplementation is sufficient to rescue CM size in kidneys lacking *Acly* in Six2-expressing cells. Five pairs of kidneys from control (CTRL) and mutant (MUT) embryos at E12.5 were cultured for 24 hours with either vehicle (a, b, c) or 10 mM sodium acetate (d, e, f). Sodium acetate prevented CM depletion observed in the mutant embryos (compare a to b and c) and promoted CM expansion in both control and mutant embryos (d to f). The immunostaining was performed using antibodies against Six2 (nephron progenitor cells, cyan), Lhx1 (nascent nephrons, red), and UB (DBA, green). The figure shows 1/5 control and 2/5 mutant kidneys. Scale bar 100 µm.
