# Supplementary material for: Acetyl-CoA is a key molecule for nephron progenitor cell pool maintenance
Source: Nat Commun. 2023 Nov 25;14:7733. doi: 10.1038/s41467-023-43513-7 (PMC10676360; doi:10.1038/s41467-023-43513-7)
Supplement: Supplementary file 2 — Description of Additional Supplementary Files [file 41467_2023_43513_MOESM2_ESM.pdf]

Description of Additional Supplementary Files

Supplementary Data 1 Differential gene expression analysis from E13.5 NPC ctrl vs YN1-treated E13.5 NPC.

Table with the genes resulting from differential expression analysis between Control (untreated) E13.5 NPC vs YN1-treated E13.5 NPC for 24h. The following columns are present: the 3 or 4-letter code representing a gene symbol (Gene symbol); The calculated logarithm (base 2) of the ratio of the gene expression levels (reported in TPM) between two conditions (LogFC), Average expression of a gene (AveExpr); Probability value, determined by the differential gene expression analyses (P-value); The magnitude of the difference in gene expression between two groups while taking into account the variability within those groups (t-value); False Detection Rate is the adjusted p-value for multiple comparisons (FDR), and the Bayes Factor (B) used to assess the evidence in favor of differential expression between two or more conditions.

Supplementary Data 2 Differential gene expression analysis from E13.5 NPC ctrl vs CHIR-treated E13.5 NPC.

Table with the genes resulting from differential expression analysis between Control (untreated) E13.5 NPC vs CHIR-treated E13.5 NPC for 24h. The following columns are present: the 3 or 4 letter code for representing a gene symbol (Gene symbol); The calculated logarithm (base 2) of the ratio of the gene expression levels (reported in TPM) between two conditions (LogFC), Average expression of a gene (AveExpr); Probability value, determined by the differential gene expression analyses (P-value); The magnitude of the difference in gene expression between two groups while taking into account the variability within those groups (t-value); False Detection Rate is the adjusted p-value for multiple comparisons (FDR), and the Bayes Factor (B) used to assess the evidence in favor of differential expression between two or more conditions.

Supplementary Data 3 Gene Ontology analysis based upon differentially expressed genes from E13.5 NPC ctrl vs YN1-treated E13.5 NPC.

Table with the GO resulting from differential gene expression analysis between Control (untreated) E13.5 NPC vs YN1-treated E13.5 NPC for 24h. The table contains the following columns: The name of a specific Gene Ontology term or category (term name); the statistical quantification of how far the gene enrichment in a GO term deviates from the mean expressed scaled (Z-score), positive Z-score indicates that more genes are associated with the term than expected by chance. In comparison, a negative Z-score indicates fewer genes than expected). The overall significance or enrichment of genes associated with a specific GO term (Combined score); The statistical correction applied to control for multiple hypothesis testing in gene ontology analysis or False Detection Rate (FDR); The probability of observing the enrichment of genes in a particular GO term by chance alone (p-value). In the GO analysis context, a smaller p-value indicates that the association between the genes and the GO term is less likely to be due to random chance; The DEG from the Ctrl vs YN1 dataset that are associated with or found within a specific GO term (overlapping genes); The collection of GO terms that are being used for enrichment analysis obtained from a specified set (genes set library); The base-10 logarithm of the p-value (log10p) and gene set status (up or downregulated in YN1 treated samples) are provided in the table.

Supplementary Data 4 Gene Ontology analysis based upon differentially expressed genes from E13.5 NPC ctrl vs CHIR-treated E13.5 NPC.

Table with the GO resulting from differential expression analysis between Control (untreated) E13.5 NPC vs CHIR-treated E13.5 NPC for 24h. The table contains the following columns: The name of a specific Gene Ontology term or category (term name); the statistical quantification of how far the gene enrichment in a GO term deviates from the mean expressed scaled (Z-score), positive Z-score indicates that more genes are associated with the term than expected by chance. In comparison, a negative Z-score indicates fewer genes than expected). The overall significance or enrichment of genes associated with a specific GO term (Combined score); The statistical correction applied to control for multiple hypothesis testing in gene ontology analysis or False Detection Rate (FDR); The probability of observing the enrichment of genes in a particular GO term by chance alone (p-value). In the GO analysis context, a smaller p-value indicates that the association between the genes and the GO term is less likely to be due to random chance; The DEG from the Ctrl vs CHIR dataset that are associated with or found within a specific GO term (overlapping genes); The collection of GO terms that are being used for enrichment analysis obtained from a specified set (genes set library); The base-10 logarithm of the p-value (log10p) and gene set status (up or downregulated in CHIR treated samples) are provided in the table.
