# Supplementary material for: Acetyl-CoA is a key molecule for nephron progenitor cell pool maintenance
Source: Nat Commun. 2023 Nov 25;14:7733. doi: 10.1038/s41467-023-43513-7 (PMC10676360; doi:10.1038/s41467-023-43513-7)
Supplement: Supplementary file 7 — Reporting Summary [file 41467_2023_43513_MOESM7_ESM.pdf]

nature portfolio

Corresponding author(s): Tortelote GG

Last updated by author(s): Oct 18, 2023

Reporting Summary

Nature Portfolio wishes to improve the reproducibility of the work that we publish. This form provides structure for consistency and transparency in reporting. For further information on Nature Portfolio policies, see our Editorial Policies and the Editorial Policy Checklist.

Statistics

For all statistical analyses, confirm that the following items are present in the figure legend, table legend, main text, or Methods section.

n/a	Confirmed	
☐	☒	The exact sample size (*n*) for each experimental group/condition, given as a discrete number and unit of measurement
☐	☒	A statement on whether measurements were taken from distinct samples or whether the same sample was measured repeatedly
☐	☒	The statistical test(s) used AND whether they are one- or two-sided<br>*Only common tests should be described solely by name; describe more complex techniques in the Methods section.*
☐	☒	A description of all covariates tested
☐	☒	A description of any assumptions or corrections, such as tests of normality and adjustment for multiple comparisons
☐	☒	A full description of the statistical parameters including central tendency (e.g. means) or other basic estimates (e.g. regression coefficient) AND variation (e.g. standard deviation) or associated estimates of uncertainty (e.g. confidence intervals)
☐	☒	For null hypothesis testing, the test statistic (e.g. *F*, *t*, *r*) with confidence intervals, effect sizes, degrees of freedom and *P* value noted<br>*Give P values as exact values whenever suitable.*
☒	☐	For Bayesian analysis, information on the choice of priors and Markov chain Monte Carlo settings
☒	☐	For hierarchical and complex designs, identification of the appropriate level for tests and full reporting of outcomes
☒	☐	Estimates of effect sizes (e.g. Cohen's *d*, Pearson's *r*), indicating how they were calculated

Our web collection on statistics for biologists contains articles on many of the points above.

Software and code

Policy information about availability of computer code

Data collection	This paper uses Nephron progenitor cells primary cells extracted from E13.5 mouse embryonic kidneys. We also used embryonic kidneys collected at several stages for the experiments shown with this paper, as described in Methods section of the main manuscript.<br>The bulk RNA sequencing series was deposited and available at NCBI GEO under the accession # GSE210937 (https://www.ncbi.nlm.nih.gov/geo/query/acc.cgi?acc=GSE210937).<br>The mass spectrometry proteomics data have been deposited to the ProteomeXchange Consortium via the PRIDE partner repository with the dataset identifier PXD045445 and 10.6019/PXD045445 (http://www.ebi.ac.uk/pride/archive/projects/PXD045445).<br>Relevant processed proteomic files have been deposited with Figshare under the following private link https://figshare.com/s/95a3dea586c92e7150de<br>Source data are provided with this paper
Data analysis	Statistical analysis and plotting were performed in R programming language (v4.1.1) and the results were plotted with the following libraries:<br>1) ggplot2 (ggplot2.tidyverse.org/);<br>2) ggpubr (github.com/kassambara/ggpubr/);<br>3) ggstatsplot (https://indrajeetpatil.github.io/ggstatsplot/).

For manuscripts utilizing custom algorithms or software that are central to the research but not yet described in published literature, software must be made available to editors and reviewers. We strongly encourage code deposition in a community repository (e.g. GitHub). See the Nature Portfolio guidelines for submitting code & software for further information.

Data

Policy information about availability of data

All manuscripts must include a data availability statement. This statement should provide the following information, where applicable:

- Accession codes, unique identifiers, or web links for publicly available datasets
- A description of any restrictions on data availability
- For clinical datasets or third party data, please ensure that the statement adheres to our policy

The bulk RNA sequencing series was deposited and available at NCBI GEO under the accession # GSE210937 (https://www.ncbi.nlm.nih.gov/geo/query/acc.cgi?acc=GSE210937).
The mass spectrometry proteomics data have been deposited to the ProteomeXchange Consortium via the PRIDE partner repository with the dataset identifier PXD045445 and 10.6019/PXD045445 (http://www.ebi.ac.uk/pride/archive/projects/PXD045445).
Relevant processed proteomic files have been deposited with Figshare under the following private link https://figshare.com/s/95a3dea586c92e7150de
Source data are provided with this paper

Research involving human participants, their data, or biological material

Policy information about studies with human participants or human data. See also policy information about sex, gender (identity/presentation), and sexual orientation and race, ethnicity and racism.

Reporting on sex and gender	NA
Reporting on race, ethnicity, or other socially relevant groupings	NA
Population characteristics	NA
Recruitment	NA
Ethics oversight	NA

Note that full information on the approval of the study protocol must also be provided in the manuscript.

Field-specific reporting

Please select the one below that is the best fit for your research. If you are not sure, read the appropriate sections before making your selection.

- [x] Life sciences
- [ ] Behavioural & social sciences
- [ ] Ecological, evolutionary & environmental sciences

For a reference copy of the document with all sections, see nature.com/documents/nr-reporting-summary-flat.pdf

Life sciences study design

All studies must disclose on these points even when the disclosure is negative.

Sample size	No statistical method was used to determine sample size. The sample size differed among experiments and it is reported throughout the manuscript and in the legend of each figure
Data exclusions	No data exclusions. However filtering technique sto select the top hits from differential gene expression analysis was employed. For intsnce for enrichment analysis the differentially expressed genes were defined with the following threshold: log fold change > 0.6, p-value < 0.05.
Replication	All experimnets were perfromed with biological and technical replicates, described in the Method section and the legend of the figures.
Randomization	NA
Blinding	NA

Reporting for specific materials, systems and methods

We require information from authors about some types of materials, experimental systems and methods used in many studies. Here, indicate whether each material, system or method listed is relevant to your study. If you are not sure if a list item applies to your research, read the appropriate section before selecting a response.

Materials & experimental systems

n/a	Involved in the study
☐	☒ Antibodies
☐	☒ Eukaryotic cell lines
☒	☐ Palaeontology and archaeology
☐	☒ Animals and other organisms
☒	☐ Clinical data
☒	☐ Dual use research of concern
☒	☐ Plants

Methods

n/a	Involved in the study
☒	☐ ChIP-seq
☒	☐ Flow cytometry
☒	☐ MRI-based neuroimaging

Antibodies

Antibodies used	1) Mouse IgG Anti-Lhx1 (Developmental Hybridoma, 4F2) (1:100 v/v) + TSA Detection (Donkey anti-Mouse IgG1 Secondary Antibody, HRP, Jackson Immuno Research™ (cat # 715-036-150) (1:100 v/v)<br>2) Rabbit IgG Anti-Acly (Proteintech, cat # 15421-1-AP) (1:200 v/v) + Donkey anti-Rabbit IgG Highly Cross-Adsorbed Secondary Antibody, Alexa Fluor™ 488 (cat # A-21202) (1:400 v/v)<br>3) Rabbit IgG Anti-Six2 (Proteintech, cat# 11562) (1:200 v/v) + Donkey anti-Rabbit IgG Highly Cross-Adsorbed Secondary Antibody, Alexa Fluor™ 555 (cat # A-31572) (1:400 v/v)<br>4) Goat IgG Anti-Wnt4 (R&D systems, cat # AF475) (1:100 v/v) + TSA Detection (Donkey anti-Goat IgG1 Secondary Antibody, HRP, Jackson Immuno Research™ (cat # 705-036-147) (1:100 v/v)<br>5) Mouse IgG Anti-Cytokeratin Pan (Sigma, cat# C2562) (1:200 v/v) + Donkey anti-Mouse IgG Highly Cross-Adsorbed Secondary Antibody, Alexa Fluor™ 647 (cat # A-311571) (1:200 v/v)<br>6) Chicken IgG Anti-GFP (Abcam, #cat ab300643) (1:200 v/v) + Donkey anti-Mouse IgG Highly Cross-Adsorbed Secondary Antibody, Alexa Fluor™ 555 (cat # A-21437) (1:400 v/v)<br>7) Fluorescent-tagged Dolichos Biflorus Agglutinin (DBA) (Vector Labs, cat# FL1031) (1:100 v/v) was used for UB-specific lectin staining.<br>8) Hoechst-33342 (ThermoFisher, cat# 62249) was used for nuclear staining with 1:10.000 v/v dilution.
Validation	all antibodies used in this manuscript are commercially available and have been validated.

Eukaryotic cell lines

Policy information about cell lines and Sex and Gender in Research

Cell line source(s)	Nephron progenitor primary cells extracted from E13.5 mouse embryonic kidneys.
Authentication	Authentication was carried out based on the tissue specificity at the collection, Cell sorting with Magnetic assisted cell sorting and trancriptome profiling of the collected cells.
Mycoplasma contamination	PCR tested
Commonly misidentified lines<br>(See ICLAC register)	NA

Animals and other research organisms

Policy information about studies involving animals; ARRIVE guidelines recommended for reporting animal research, and Sex and Gender in Research

Laboratory animals	CD1 outbred mice were acquired from Charles River Laboratories and bred in-house at our SPF facility. The mice were kept in a standard microisolator cage. We use a 12-hour lights on/12-hour lights off cycle. The temperature was kept between 70-72°F (~21°C) with 40-60% humidity. All females utilized for this study were between 5 and 8 weeks old, and only litters of a first pregnancy were collected. Transgenic or wild-type male mice utilized here ranged from 6 weeks old to 6 months old. For our studies, sex was not considered a biological variable. There is no evidence in the literature to support that sex is an important biological variable in E12.5 E13.5 and P0 mouse offspring regarding the impact of metabolism on kidney development, and thus information regarding sex as a biological variable has not been collected. Acly floxed animals were acquired from the Jackson Laboratory, stock #030772. Six2GFP-Cre transgene mice were acquired from the Jackson Laboratory, stock #009606.
Wild animals	NA
Reporting on sex	For our studies, sex was not considered a biological variable. There is no evidence in the literature to support that sex is an important biological variable in E12.5 E13.5 and P0 mouse offspring regarding the impact of metabolism on kidney development, and thus information regarding sex as a biological variable has not been collected.
Field-collected samples	NA

Ethics oversight	Animal protocols utilized in this study were approved by and in strict adherence to guidelines established by the Tulane University Institutional Animal Care and Use Committee.

Note that full information on the approval of the study protocol must also be provided in the manuscript.

Plants

Seed stocks	NA
Novel plant genotypes	NA
Authentication	NA
